# Bioproduction of methylated phenylpropenes and isoeugenol in *Escherichia coli*

**DOI:** 10.1016/j.mec.2024.e00237

**Published:** 2024-05-15

**Authors:** Jeremy Chua, Erik K.R. Hanko, Andrew Yiakoumetti, Ruth A. Stoney, Jakub Chromy, Kris Niño G. Valdehuesa, Katherine A. Hollywood, Cunyu Yan, Eriko Takano, Rainer Breitling

**Affiliations:** Manchester Institute of Biotechnology, Faculty of Science and Engineering, University of Manchester, 131 Princess Street, Manchester, M1 7DN, United Kingdom

**Keywords:** Phenylpropene, Biosynthesis, *Escherichia coli*, SAM-Dependent *O*-Methyltransferase, Methyleugenol

## Abstract

Phenylpropenes are a class of natural products that are synthesised by a vast range of plant species and hold considerable promise in the flavour and fragrance industries. Many *in vitro* studies have been carried out to elucidate and characterise the enzymes responsible for the production of these volatile compounds. However, there is a scarcity of studies demonstrating the *in vivo* production of phenylpropenes in microbial cell factories. In this study, we engineered *Escherichia coli* to produce methylchavicol, methyleugenol and isoeugenol from their respective phenylacrylic acid precursors. We achieved this by extending and modifying a previously optimised heterologous pathway for the biosynthesis of chavicol and eugenol. We explored the potential of six *S*-adenosyl l-methionine (SAM)-dependent *O-*methyltransferases to produce methylchavicol and methyleugenol from chavicol and eugenol, respectively. Additionally, we examined two isoeugenol synthases for the production of isoeugenol from coniferyl acetate. The best-performing strains in this study were able to achieve titres of 13 mg L^−1^ methylchavicol, 59 mg L^−1^ methyleugenol and 361 mg L^−1^ isoeugenol after feeding with their appropriate phenylacrylic acid substrates. We were able to further increase the methyleugenol titre to 117 mg L^−1^ by supplementation with methionine to facilitate SAM recycling. Moreover, we report the biosynthesis of methylchavicol and methyleugenol from l-tyrosine through pathways involving six and eight enzymatic steps, respectively.

## Highlights

• Computational retrobiosynthesis software identified successful enzyme candidates.
• 59 mg L^−1^ methyleugenol and 13 mg L^−1^ methylchavicol produced from 3 mM fed substrate.
• Methyleugenol production titres doubled by supplementing cultures with methionine.
• Methyleugenol and methylchavicol produced from central metabolites in *E. coli.*

## 1 Introduction

Phenylpropenes are a class of volatile phenylpropanoids that are produced naturally across the plant kingdom (Humphreys et al., 2002). These compounds play important roles in plant physiology, defence against herbivores and attracting pollinators (Gang et al., 2001). Phenylpropenes have also been reported to be the main constituents in essential oils and spices (Chaieb et al., 2007; Jirovetz et al., 2006). Due to their properties, phenylpropenes have been acknowledged as desirable compounds and are highly sought after within the food and cosmetic industries (Han et al., 2013). Namely, chavicol, eugenol, isoeugenol and their methylated derivatives, are industrially relevant phenylpropenes widely used in food, perfumes, soaps, and detergents (Ismaiel et al., 2016; Program, 2000; Koeduka, 2014).

**Fig. 1:**
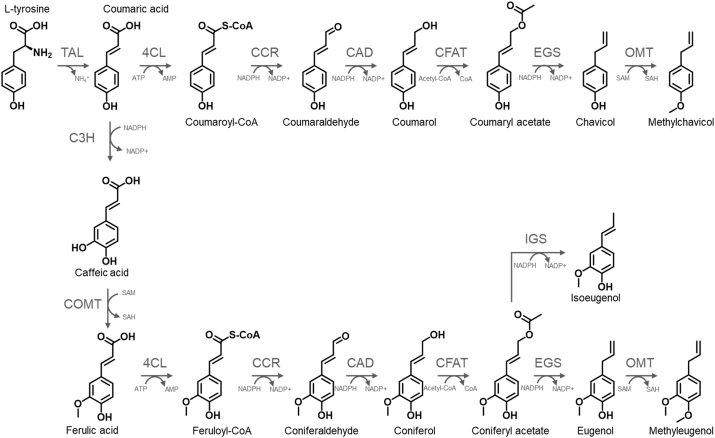
Phenylpropene biosynthesis pathway via CoA-dependent activation of the phenylacrylic acid substrate. Enzyme abbreviations and EC numbers: TAL, tyrosine ammonia-lyase (EC: 4.3.1.25); C3H, coumarate 3-hydroxylase (EC: 1.14.13.-); COMT, caffeic acid 3-*O*-methyltransferase (EC: 2.1.1.68); 4CL, 4-coumarate-CoA ligase (EC: 6.2.1.12); CCR, cinnamoyl-CoA reductase (EC: 1.2.1.44); CAD, cinnamyl-alcohol dehydrogenase (EC: 1.1.1.195); CFAT, coniferyl alcohol acyltransferase (EC: 2.3.1.84); EGS, eugenol synthase (EC: 1.1.1.318); IGS, isoeugenol synthase (EC: 1.1.1.319); OMT, (iso)eugenol *O*-methyltransferase (EC: 2.1.1.146).

The shikimate pathway provides the entry point of the phenylpropanoid metabolic pathway through the production of the aromatic amino acids tyrosine and phenylalanine (Biała et al., 2018). These compounds are the key building blocks towards phenylpropene production. For example, l-tyrosine can be converted into *p-*coumaric acid via tyrosine ammonia-lyase (TAL). From there, *p-*coumaric acid can be further converted into *trans-*ferulic acid, and these phenylacrylic acid precursors can be converted into a wide range of phenylpropanoids. Methylchavicol and methyleugenol are naturally synthesised through the methylation of their phenylpropene precursors chavicol and eugenol, respectively (Hahlbrock et al., 1989). This reaction is catalysed by *S*-adenosyl l-methionine (SAM)-dependent (iso)eugenol *O*-methyltransferases (OMTs). Additionally, isoeugenol can be produced through the reduction of coniferyl acetate by an NADPH-dependent reductase, isoeugenol synthase (IGS) (Fig. 1) (Koeduka, 2014, 2018).

The plethora of applications and the value of these compounds have generated considerable interest towards the elucidation of the phenylpropene biosynthesis pathways in plants, specifically focusing on isolating and characterising the genes and enzymes involved at each step of the pathways with the ultimate aim of heterologous phenylpropene production in engineered microbial systems (Wang et al., 1997; Koeduka et al., 2009; Yauk et al., 2015; Yahyaa et al., 2019). Although extensive research has been conducted to characterise the individual enzymes responsible for the biosynthesis of phenylpropenes *in vitro*, there are only a few studies that simultaneously express these enzymes in recombinant microorganisms to produce these compounds.

Kim and colleagues were the first to demonstrate the microbial production of chavicol and eugenol from their monolignol precursors, coumarol and coniferol, respectively (Kim et al., 2014). The authors utilised enzymes that naturally catalyse the conversion of coniferol into eugenol and isoeugenol in *Larrea tridentata*. They co-expressed cinnamyl alcohol acyltransferase (LtCAAT1) with either an allylphenol synthase (LtAPS1) or a propenyl synthase (LtPPS1), in *Escherichia coli*. The acyltransferase LtCAAT1 catalysed the conversion of coniferol into coniferyl acetate. Following this, the phenylpropene synthases, LtAPS1 and LtPPS1, catalysed the subsequent reduction to eugenol and isoeugenol, respectively (Vassão et al., 2007).

More recently, the production of eugenol and chavicol from their phenylacrylic acid precursors, *trans-*ferulic acid and *p-*coumaric acid, respectively, has been demonstrated in *E. coli* (Robinson et al., 2020). The authors utilised the natural phenylpropene biosynthetic pathway in plants (Fig. 1) whereby the phenylacrylic acids are activated as Coenzyme A (CoA) thioesters before the subsequent double reduction to their respective monolignols. The monolignols are then converted to eugenol and chavicol. Using this approach, the authors engineered an *E. coli* strain that produced 0.21 mM (28 mg L^−1^) chavicol and 0.62 mM (102 mg L^−1^) eugenol when supplemented with 3 mM *p-*coumaric acid and 3 mM *trans-*ferulic acid, respectively (Robinson et al., 2020).

Building on this, an alternative pathway was explored to produce the monolignol intermediates, coumarol and coniferol, from their respective phenylacrylic acid precursors (Hanko et al., 2023). The authors employed a carboxylic acid reductase (CAR) that directly reduces phenylacrylic acids to their aldehyde form, bypassing the need to be activated to their CoA thioesters and thereby shortening the pathway by one catalytic step. As a result, the authors produced 1.66 mM (223 mg L^−1^) chavicol and 1.61 mM (264 mg L^−1^) eugenol when supplemented with 3 mM *p-*coumaric acid and 3 mM *trans-*ferulic acid, respectively.

In an alternate approach, eugenol and chavicol were produced from simple sugar sources (glucose and glycerol) in a tripartite *E. coli* co-culture where the eugenol biosynthesis pathway from central carbon metabolism was split into three distinct modules present in each respective strain (Brooks et al., 2023). The three modules compartmentalised the coumaric acid, ferulic acid and eugenol biosynthesis pathways (Fig. 1) and were optimised in a 1:3:1 ratio to achieve their maximum titres. Through this approach, the authors were able to produce up to 0.4 mM (66 mg L^−1^) eugenol without additional supplementation of intermediary compounds. Similarly, enzyme promiscuity was leveraged to enable the production of chavicol.

In this study, we aim to extend the previously described CAR-dependent phenylpropene biosynthesis pathway to demonstrate the microbial production of methylchavicol and methyleugenol as well as modify this pathway to produce isoeugenol. Here, the microbial production of methylchavicol, methyleugenol and isoeugenol is achieved using a two-module pathway in *E. coli*. The first module utilises the CAR-dependent biosynthesis pathway as previously described (Hanko et al., 2023), catalysing the conversion of phenylacrylic acid substrates into monolignol intermediates. The second module builds on a previously described phenylpropene production pathway from monolignols (Robinson et al., 2020).

Six OMT candidates were selected and individually inserted into the pathway to facilitate the methylation of chavicol and eugenol to methylchavicol and methyleugenol, respectively. Additionally, for the production of isoeugenol, the EGS gene was substituted with one of the two selected IGS gene candidates. Furthermore, we demonstrate the production of methylchavicol and methyleugenol directly from l-tyrosine by including a third module that comprises previously optimised phenylacrylic acid production pathways on a separate plasmid (Robinson et al., 2020; Dunstan et al., 2020). In doing so, this enabled the production of methylchavicol and methyleugenol directly from l-tyrosine through metabolic pathways of six and eight steps, respectively.

## 2 Materials and methods

### 2.1 Bacterial strains

Routine cloning, plasmid propagation and phenylpropene biosynthesis were performed using *E. coli* NEB5α cells (New England Biolabs). Bacterial strains were grown in Lysogeny Broth (LB, Formedium) or on LB-agar (Formedium) supplemented with the appropriate antibiotics for plasmid selection unless stated otherwise. The standard antibiotic concentrations used were: 100 μg mL^−1^ carbenicillin, 50 μg mL^−1^ kanamycin, and 34 μg mL^−1^ chloramphenicol. All strains used and generated in this study are listed in Supplementary Table S1.

### 2.2 Enzyme selection

The online enzyme selection tool Selenzyme (Carbonell et al., 2018) complemented with a manual literature research was used to identify suitable enzyme candidates for the *O*-methylation of chavicol and eugenol, and for the synthesis of isoeugenol from coniferyl acetate.

For the Selenzyme search process, both reaction EC number-based queries (EC 2.1.1.146 or EC 2.1.1.279 for chavicol and eugenol methylation; EC 1.1.1.319 for isoeugenol synthesis) and SMILES-based queries were used with default Selenzyme settings, and only reaction similarity and UniProt protein evidence scores were considered, so as not to penalise against enzymes from distant organism groups.

For the final selection of enzymes, evidence of their successful recombinant expression in *E. coli*, evidence of catalytic activity with the desired substrates and products, and availability of kinetic parameters, were taken into consideration. The enzyme candidates used in this study are listed in Supplementary Table S2.

### 2.3 Plasmid assembly

Oligonucleotide primers were synthesised by Integrated DNA Technologies (IDT) and the primer sequences can be found in Supplementary Table S3. Gene parts were designed using PartsGenie (Swainston et al., 2018), optimised for *E. coli* codon usage and subsequently synthesised by Twist Bioscience. The sequences of the synthesised DNA fragments can be found in Supplementary Table S4. Gene parts and the intergenic region harbouring the *trc* promoter were PCR-amplified using the appropriate primers and gel-purified using the Zymoclean Gel DNA Recovery Kit (Zymo Research).

The vector backbone, SBC009876, used for the plasmid construction was kindly provided by Robinson et al. (2020). All plasmids assembled in this study were built by digesting the vector backbone using BamHI (NEB) or a combination of BamHI, EcoRI (NEB) and HindIII (NEB), and subsequent gel purification. Plasmids were constructed manually by HiFi DNA Assembly (NEB). A detailed description of the plasmid assemblies can be found in the Supplementary Methods.

Plasmids were transformed into chemically competent *E. coli* NEB5α and grown on LB-agar supplemented with the appropriate antibiotics. Plasmid DNA was obtained using the QIAprep Spin Miniprep Kit (Qiagen) and verified via Sanger sequencing (Eurofins Genomics). The plasmids used and assembled in this study can be found in Supplementary Table S5. All kits were used in accordance with the manufacturer's instructions.

### 2.4 Biosynthesis of phenylpropenes

The production assay used in this study was largely adopted from Robinson et al. (2020). Briefly, individual colonies of freshly transformed cells were used to inoculate 1 mL of phosphate-buffered Terrific Broth (TBP, Formedium), supplemented with 0.4% glycerol (w/v) and the relevant antibiotics. The seed cultures were grown in 96-deepwell plates (DWP) sealed with breathable plate seals. The cultures were incubated overnight at 30 °C and 80% humidity with shaking at 850 rpm. The main culture was prepared by diluting the seed culture to a final OD_600nm_ of 0.02 in 1.5 mL of fresh TBP medium supplemented with 0.4% glycerol (w/v) and the relevant antibiotics in a 96-DWP. The main culture was returned to the shaker-incubator and left to grow until an OD_600nm_ of 1.0–2.0 was reached. At this point, 1 mL of each culture was transferred to a 20-mL headspace vial and overlaid with 0.5 mL of 2,2,4-trimethylpentane (TMP, Sigma-Aldrich) containing 0.005% (v/v) *sec*-butylbenzene (*sec*-B, Sigma-Aldrich). Where appropriate, pathway substrates were added to final concentrations of 3 mM and cultures were induced by adding isopropyl β-D-1-thiogalactopyranoside (IPTG) to a final concentration of 100 μM. When required, l-methionine was added to the cultures to a final concentration of 10 mM. Vials were sealed with gas-tight screw caps and placed in a shaker-incubator for 24 h at 30 °C and 200 rpm. The compounds used either as substrates or calibration standards are listed in Supplementary Table S6.

### 2.5 Quantification of target compounds

Due to the different physical and chemical properties of the target compounds, precursors and selected intermediates measured in this study, we used a range of analytical instruments to quantify the individual pathway metabolites. l-Tyrosine was quantified using liquid chromatography with tandem mass spectrometry (LC-MS/MS) analysis, phenylacrylic acids were quantified using ultra high performance liquid chromatography with diode array detection (UPLC-DAD) analysis, and phenylpropenes were quantified using gas chromatography (GC-MS) analysis. Samples were prepared as previously reported (Robinson et al., 2020).

Following incubation for 24 h, the cultures (organic overlay included) were transferred to microcentrifuge tubes (2 mL) and centrifuged (10,000×*g*, 1 min) to ensure complete phase separation. For UPLC and LC-MS/MS analysis, 100 μL of the aqueous phase was transferred to a 96-well microtitre plate and quenched with an equal volume of LC-MS grade methanol (100%, Honey-Well), vortexed and stored at −80 °C overnight or until analysis by UPLC-DAD or LC-MS/MS. Samples were thawed, centrifuged (2700×*g*, 10 min) and diluted five-fold in LC-MS grade water (Honey-Well). Further dilutions, if required, were done using 10% methanol.

The remainders of the cultures were centrifuged (10,000×*g*, 3 min) to pellet the cells. The separated culture medium and organic layer were individually recovered and prepared for GC-MS analysis. One part culture medium was added into 19 parts of TMP containing 0.005% (v/v) *sec-*B and the organic layers were diluted 20-fold using TMP containing 0.005% (v/v) *sec-*B. Following this, anhydrous Na_2_SO_4_ (Fisher Scientific) was added to the samples, vortexed vigorously to remove residual water and centrifuged (10,000×*g*, 1 min). 100 μL of the dry samples were transferred to amber GC-MS vials (Agilent Technologies) for analysis.

The UPLC-DAD targets, *p-*coumaric acid and *trans-*ferulic acid, were quantified using an Agilent Technologies 1290 Infinity II UHPLC System equipped with a 1290 Infinity II Multisampler, a 1290 Infinity II Multicolumn Thermostat and a 1290 Infinity II Diode Array Detector. The LC-MS/MS target, l-tyrosine, was quantified using a UHPLC chromatography system (Waters Acquity UPLC H-class) coupled to a Xevo TQ-S triple-quadrupole mass spectrometer (Waters) equipped with an electrospray ionisation source (Waters). The GC-MS targets, chavicol, eugenol and their methyl-derivatives, were quantified using an Agilent Technologies 5975 MSD coupled to a 7890B Gas Chromatograph with an Agilent Technologies 7693 autosampler. Details of the chromatography parameters used are described in the Supplementary Methods.

## 3 Results and discussion

### 3.1 Enzyme selection

For the biosynthesis of methylchavicol and methyleugenol from chavicol and eugenol, respectively, we investigated a total of six *O-*methyltransferase (OMT) enzymes, including EOMT1 from *Ocimum basilicum* (Gang et al., 2002), IEMT1 from *Clarkia breweri* (Wang et al., 1998), RcOMT1 from *Rosa chinensis* var. *spontanea* (Wu et al., 2003), AIMT1 from *Pimpinella anisum* (Koeduka et al., 2009), as well as MdoOMT1aΔ and MdoOMT1b from *Malus domestica* (Yauk et al., 2015). To produce isoeugenol from coniferyl acetate, we examined two isoeugenol synthase (IGS) enzymes, namely PhIGS1 from *Petunia hybrida* and CbIGS1 from *Clarkia breweri* (Koeduka et al., 2008).

All enzyme candidates were identified using the enzyme selection tool Selenzyme (Carbonell et al., 2018). These enzymes have previously been expressed recombinantly in *E. coli* and their catalytic activities have been extensively characterised *in vitro*. However, it should be noted that the MdoOMT1aΔ and MdoOMT1b enzymes were identified solely through literature research and had been successfully expressed recombinantly only in *Nicotiana benthamiana* as no soluble proteins had been obtained when expressed in *E. coli* and yeast (Yauk et al., 2015).

Out of the six selected OMT enzymes, ObEOMT1, RcOMT1, PaAIMT1 MdoOMT1aΔ and MdoOMT1b have been described to exhibit enzymatic activity towards both chavicol and eugenol. MdoOMT1aΔ is a truncated version of MdoOMT1a and has been shown to exhibit a higher catalytic activity *in vitro* (Yauk et al., 2015). MdoOMT1a itself is highly similar to MdoOMT1b, differing by only three amino acids. In contrast, CbIEMT1 was shown to require a methoxy group at the *meta*-position; therefore, it was reported to only *O*-methylate eugenol but not chavicol (Wang et al., 1998) and was excluded from the selection of OMT enzyme candidates for methylchavicol production. Both selected IGS enzymes, PhIGS1 and CbIGS1, were shown to be extremely selective and only catalyse the formation of isoeugenol and not eugenol (Koeduka et al., 2008). Considering all these enzymes have been catalytically characterised only individually and *in vitro*, we decided to comparatively evaluate their capability to produce their respective phenylpropene products in *E. coli*.

### 3.2 Biosynthesis of methylated phenylpropenes from phenylacrylic acid substrates

One of the objectives of this study was to produce methylchavicol and methyleugenol from their respective phenylacrylic acid precursors in *E. coli.* Previous studies have reported the production of the phenylpropenes, chavicol and eugenol, from *p-*coumaric acid and *trans-*ferulic acid, respectively, by utilising either a CoA-dependent pathway (Robinson et al., 2020; Brooks et al., 2023) or a CAR-dependent pathway (Hanko et al., 2023). The best-performing CAR-dependent pathway (encoded by plasmid SBC015869) yielded 1.66 mM (223 mg L^−1^) chavicol and 1.61 mM (264 mg L^−1^) eugenol from 3 mM *p-*coumaric acid and 3 mM *trans-*ferulic acid (Hanko et al., 2023), representing a marked increase in production titres compared to the CoA-dependent pathway employed by Robinson et al. (2020). As the CAR-dependent pathway produced higher titres of the direct precursors of methylchavicol and methyleugenol, we attempted to expand on this pathway to *O-*methylate chavicol and eugenol.

The CAR-dependent pathway built by Hanko and colleagues (Hanko et al., 2023) was conceptualised as two modules, with each module being encoded on a separate plasmid. The first module (SBC015869) encoded three enzymes: CAR from *Segniliparus rugosus* that reduces the phenylacrylic acids to their corresponding aldehydes; Sfp from *Bacillus subtilis* required for CAR activity; and coniferyl alcohol dehydrogenase (CAD) from *Medicago sativa*, which reduces the aldehydes to their respective monolignols (Hanko et al., 2023). The second module (encoded by plasmid SBC009876) was optimised to convert coumarol into chavicol as well as coniferol into eugenol (Robinson et al., 2020). It is composed of two enzymes: coniferyl alcohol acyltransferase (CFAT) from *Petunia hybrida* and eugenol synthase (EGS) from *Ocimum basilicum*.

**Fig. 2:**
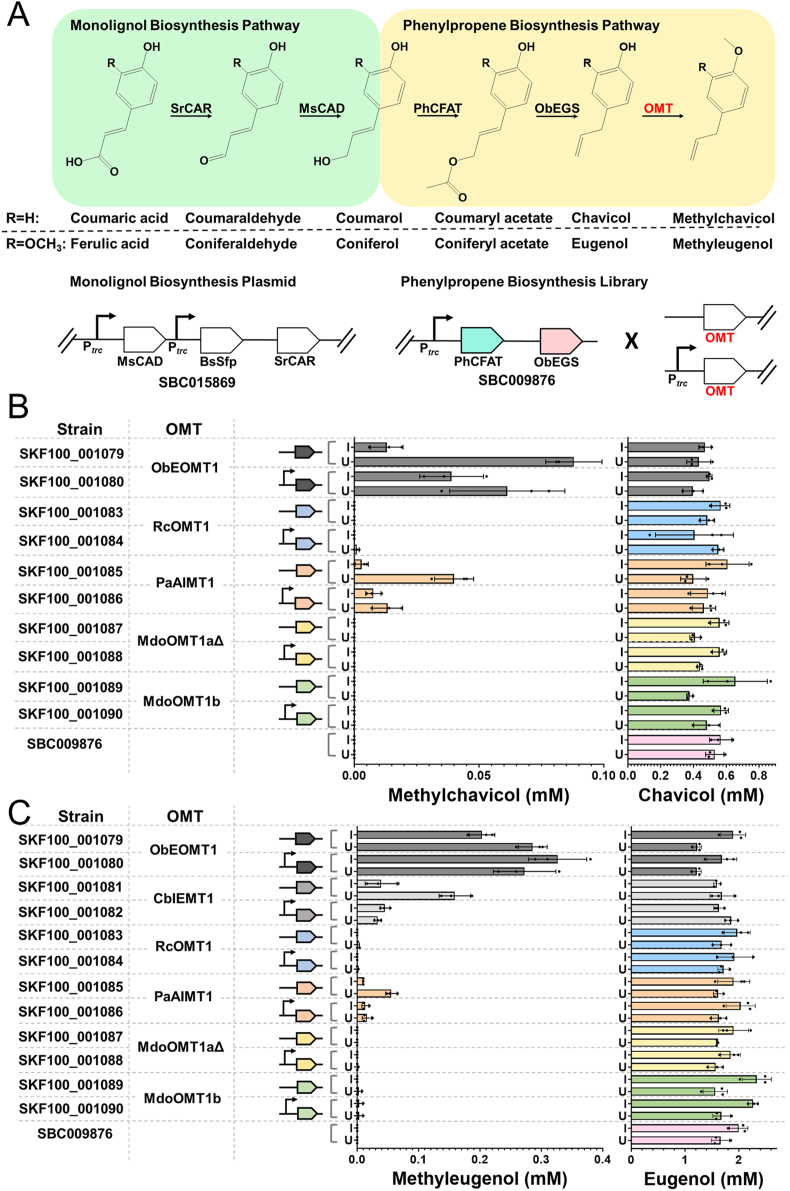
Microbial biosynthesis of phenylpropenes by whole-cell bioconversion of phenylacrylic acids. **A** Monolignol (green) and phenylpropene (yellow) biosynthesis pathways (constructs not drawn to scale). Enzyme abbreviations: SrCAR (*Segniliparus rugosus* carboxylic acid reductase); MsCAD (*Medicago sativa* cinnamyl alcohol dehydrogenase); PhCFAT (*Petunia hybrida* coniferyl alcohol acyltransferase); ObEGS (*Ocimum basilicum* eugenol synthase); OMT (*O*-methyltransferase). Plasmid SBC015869 (Hanko et al., 2023) was used for the biosynthesis of monolignols from phenylacrylic acids. The combinatorial plasmid library for phenylpropene production was constructed by inserting OMT gene candidates, either with or without a separate promoter, into plasmid SBC009876 (Robinson et al., 2020). **B** Library of strains tested for methylchavicol (left bar chart) and chavicol (right bar chart) production. **C** Library of strains tested for methyleugenol (left bar chart) and eugenol (right bar chart) production. Different OMT gene candidates used in each strain are represented by individual colours and the presence of the *trc* promoter is denoted by the arrow. Strain SBC009876 does not possess any OMT gene and was used as a control strain. All measurements were taken 24 h after induction and addition of the appropriate substrates. All experiments were performed using biological triplicates and error bars are representative of the standard deviations of these triplicates. Strains were grown in the absence (U) and presence (I) of IPTG.

To achieve the production of methylchavicol and methyleugenol, we constructed a combinatorial plasmid library by inserting the selected SAM-dependent OMT candidates (Supplementary Table S2) into the plasmid encoding CFAT and EGS (SBC009876). These SAM-dependent OMTs catalyse the *para-O* methylation of the phenol ring (Wang et al., 1997). Furthermore, to optimise their expression, each gene was cloned into the plasmid either with an upstream promoter region or without (Fig. 2A). Candidate OMT genes inserted without an upstream promoter region were expressed in an operon with CFAT and EGS. As a result, for each enzyme candidate, there were two different plasmid configurations, thus expanding the methylchavicol and methyleugenol combinatorial library to 10 (Figs. 2B) and 12 (Fig. 2C) plasmids, respectively.

Co-transforming *E. coli* with the combinatorial library of plasmids containing the OMT candidates with the plasmid encoding the CAR-dependent monolignol pathway (SBC015869 (Hanko et al., 2023)) should enable the production of methylchavicol and methyleugenol from *p-*coumaric acid and *trans-*ferulic acid, respectively. The resulting strains were grown in rich media for 24 h in both induced and uninduced conditions after supplementation with 3 mM of phenylacrylic acid substrates. The expression of the operon encoding CFAT and EGS as well as inserted OMT candidate genes is under the control of a *trc* promoter, a strong promoter associated with leaky transcription (Rosano et al., 2014). In order to balance maximising product yield with metabolic burden, the strains were tested in the presence and absence of IPTG (Tegel et al., 2011). In both induced and uninduced conditions, methylchavicol was detected in four out of the 10 strains tested (Fig. 2B), and methyleugenol was detected in six out of the 12 strains tested (Fig. 2C).

Across the enzyme candidates used in the study, EOMT1 from *O. basilicum* was reported to have the highest substrate affinity to both chavicol and eugenol *in vitro* (Wang et al., 1997, 1998; Koeduka et al., 2009; Yauk et al., 2015; Gang et al., 2002; Wu et al., 2003). In agreement with this, the highest titres obtained for methylchavicol and methyleugenol in this study were observed in strains carrying the EOMT1 gene expressed in an operon with CFAT and EGS, producing 0.09 mM (13.3 mg L^−1^) methylchavicol from 3 mM *p-*coumaric acid in the absence of IPTG (Fig. 2B). By contrast, the highest methyleugenol titre achieved was observed in strain SKF100_001080, which carries the EOMT1 gene with an upstream *trc* promoter, producing 0.33 mM (58.8 mg L^−1^) methyleugenol from 3 mM *trans-*ferulic acid in the presence of IPTG (Fig. 2C). Notably, strains carrying the genes encoding MdoOMT1aΔ (SKF100_001087 and SKF100_001088) and MdoOMT1b (SKF100_001089 and SKF100_001090) did not produce detectable amounts of methylchavicol (Fig. 2B) and only trace amounts of methyleugenol (Fig. 2C), which might be the result of protein insolubility as previously reported (Yauk et al., 2015). Generally, higher titres of methylchavicol and methyleugenol were observed in the absence of IPTG. This could suggest a lower metabolic burden placed on the cells due to decreased gene expression in the absence of the inducer IPTG, ultimately leading to higher titres.

### 3.3 Improving methyleugenol titres

No *trans-*ferulic acid or *p-*coumaric acid was detectable in strains SKF100_001079–001090 after 24 h; this suggested the complete consumption of these phenylacrylic acid substrates by the cells carrying the phenylpropene biosynthesis pathway. On this basis, we hypothesised that an increase in substrate fed to the cells would result in the increased production of the downstream intermediates and, more importantly, an increase in phenylpropene titres. We tested this hypothesis by increasing the amount of fed *trans-*ferulic acid substrate to 6 mM and 12 mM and recorded the production of methyleugenol in the corresponding best-producing strain, SKF100_001080.

**Fig. 3:**
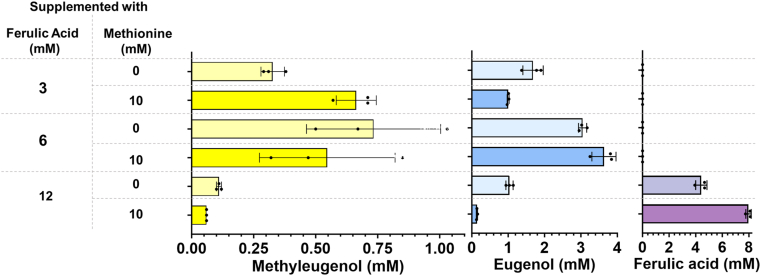
Methyleugenol and eugenol titres reported in the best-producing methyleugenol strain, SKF100_001080, in conditions of increasing *trans-*ferulic acid concentrations with and without additional 10 mM l-methionine supplementation. Methyleugenol, eugenol and unconverted *trans-*ferulic acid concentrations measured after 24 h after addition of the appropriate substrates. All strains were grown in the presence of IPTG, and measurements were taken 24 h after induction and addition of the appropriate substrates. All experiments were performed using biological triplicates and error bars are representative of the standard deviations of these triplicates.

When 6 mM *trans-*ferulic acid was added, we observed an increase in methyleugenol titres to 0.73 mM (130.1 mg L^−1^), which translates roughly to a two-fold increase when compared to that of the same strain fed with only 3 mM *trans-*ferulic acid (Fig. 3A). Although methyleugenol titres had improved, we still experienced an accumulation of eugenol as only ∼19% of total eugenol produced was converted into methyleugenol, suggesting a bottleneck in the *O-*methylation of eugenol to methyleugenol. When 12 mM *trans-*ferulic acid was supplemented into the cultures, there was a significant decrease in methyleugenol production to 0.33 mM (58.8 mg L^−1^) (Fig. 3A and B) and up to 8.1 mM of unconverted *trans-*ferulic acid remained in the media after 24 h (not shown). At the same time, the cell density observed after 24 h was lower than that observed when lower concentrations of *trans-*ferulic acid were supplemented (Supplementary Fig. S1). The observed results could be explained by growth inhibition from *trans-*ferulic acid, since this is a phenolic acid and phenolic acids are known to inhibit the growth of *E. coli* (Barthelmebs et al., 2001).

Additionally, in the best-performing strains for methylchavicol (SKF100_001079) and methyleugenol (SKF100_001080) production, there was a significant amount of phenylpropene precursor that remained unconverted. Only ∼7% of total chavicol and ∼16% of total eugenol produced were converted into methylchavicol and methyleugenol, respectively. These low conversion rates point to a bottleneck in the methylation reaction. SAM-dependent OMTs require SAM as a cofactor to donate its methyl group for the production of the target compound (Loenen, 2006). SAM is produced within the cells through the endogenous conversion of methionine by methionine adenosyltransferase encoded by *metK* (Li et al., 1998). The depletion of SAM and methionine resources in the culture has been reported to be a limiting factor in the conversion of protocatechuate to vanillate catalysed by a SAM-dependent catechol OMT (Kunjapur et al., 2016). We, therefore, hypothesised that the main contributing factor towards the low conversion rates of chavicol and eugenol into methylchavicol and methyleugenol was the limited availability of SAM in the cultures.

In this regard, we supplemented cultures of strain SKF100_001080 with 10 mM l-methionine and 3 mM *trans-*ferulic acid upon induction. We observed an approximately two-fold increase in methyleugenol production to 0.66 mM (117.6 mg L^−1^) compared to the same strain grown in the absence of l-methionine (0.33 mM [58.8 mg L^−1^]) (Fig. 3B). This was accompanied by a decrease in eugenol titres to 1.0 mM (164.2 mg L^−1^). The respective changes in the methyleugenol and eugenol titres observed after the addition of l-methionine translate to about a 2.5-fold increase in conversion of eugenol to methyleugenol. These results are in line with a previous study that reported a two-fold increase in vanillate titres from protocatechuate observed in cultures after the supplementation of 10 mM l-methionine (Kunjapur et al., 2016).

We expected to observe the same trend reflecting this increase in methyleugenol titres when l-methionine was supplemented to a final concentration of 10 mM in cultures of strain SKF100_001080 along with *trans-*ferulic acid to final concentrations of both 6 mM and 12 mM. However, under these conditions, we did not observe an increase in methyleugenol titres as only 0.55 mM (98.0 mg L^−1^) and 0.06 mM (10.7 mg L^−1^) methyleugenol was produced when l-methionine was added with 6 mM and 12 mM *trans-*ferulic acid, respectively (Fig. 3B). As mentioned earlier, high concentrations of *trans-*ferulic acid can be inhibitory towards *E. coli* growth (Barthelmebs et al., 2001). This can be seen in our data, as the final cell density of cultures supplemented with 12 mM *trans-*ferulic acid was more than 6-fold lower than that of cultures supplemented with 3 mM and 6 mM *trans-*ferulic acid (Supplementary Fig. S1); this reduced growth was accompanied by a significant decrease in methyleugenol production (Fig. 3).

It should be noted that cultures supplemented with both 6 mM *trans-*ferulic acid and 10 mM l-methionine also showed a roughly 2-fold decrease in final cell density (Supplementary Fig. S1); however, methyleugenol production remained almost unchanged (Fig. 3). This decrease in final cell density could be explained by the increased concentration of SAM as a result of l-methionine supplementation. *S*-adenosyl l-homocysteine (SAH) is a by-product of SAM-dependent methylation reactions after SAM donates its methyl group to the recipient compound (Zhang et al., 2016) and has been reported to have inhibitory growth effects on *E. coli* (Roe et al., 2002) which could explain the decrease in final cell density observed in these cultures.

Apart from SAM and methionine availability, SAM-dependent methylation reactions are influenced by the inhibitory effect exerted by *S*-adenosyl l-homocysteine (SAH) (Zhang et al., 2016). Studies have shown that its inhibitory effects can be subverted through genetic engineering by promoting SAH recycling back into SAM (Lee et al., 2019; Wei et al., 2021; Liu et al., 2022). For example, vanillate production titres from protocatechuate were increased by 25% when *mtn* and *luxS* were overexpressed in the *E. coli* host (Kunjapur et al., 2016). These enzymes are responsible for the conversion of SAH into *S*-ribosyl l-homocysteine (SRH) and subsequently to l-homocysteine, respectively. Homocysteine can then be converted into l-methionine, preventing the intracellular accumulation of SAH while simultaneously replenishing the methionine and SAM pools (Zhang et al., 2021). To this end, this approach could be utilised in future work to genetically engineer an *E. Coli* strain to promote SAH recycling, circumvent its inhibitory effects and increase production titres. Additionally, further strain development efforts could be performed in coordination with scale-up exercises in fed-batch fermentations to limit the amount of *trans*-ferulic acid present in the culture medium and to enhance the production of eugenol and methyleugenol.

### 3.4 Biosynthesis of methylated phenylpropenes from l-tyrosine

The phenylacrylic acids – *p-*coumaric acid and *trans-*ferulic acid – used thus far in this study as substrates, have been previously produced in *E. coli* from l-tyrosine (Robinson et al., 2020; Dunstan et al., 2020). The production of *p-*coumaric acid from l-tyrosine was achieved by the expression of a single enzyme, tyrosine ammonia-lyase (TAL). When *Flavobacterium johnsoniae* TAL (FjTAL) was expressed in *E. coli*, 0.34 g L^−1^ *p-*coumaric acid was produced, and 1.02 g L^−1^ *p-*coumaric acid was produced when cultures were further supplemented with 3 mM l-tyrosine (Robinson et al., 2020). The production of *trans-*ferulic acid from l-tyrosine was reported to be achieved by the expression of FjTAL together with coumarate 3-hydroxylase from *Saccharothrix espanensis* (SeC3H) and caffeate 3-*O*-methyltransferase from *Populus kitakamiensis* (PkCOMT). When expressed in *E. coli* and supplemented with 3 mM l-tyrosine, up to 198 mg L^−1^ *trans-*ferulic acid was produced (Dunstan et al., 2020).

**Fig. 4:**
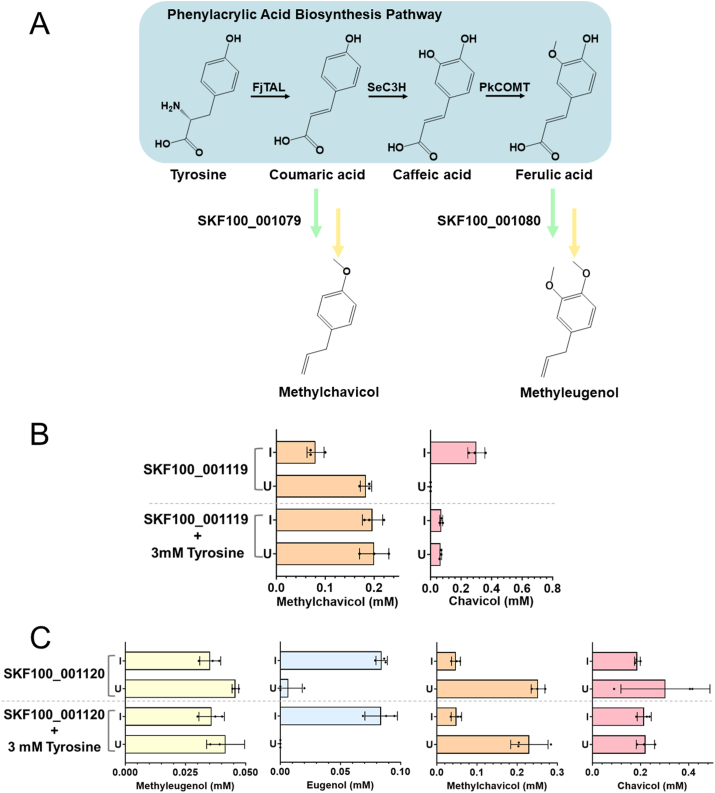
Extending microbial biosynthesis of methylated phenylpropenes from l-tyrosine. **A** The *p-*coumaric acid biosynthesis pathway (SBC007589 (Robinson et al., 2020)) or the *trans-*ferulic acid biosynthesis pathway (SBC010695 (Dunstan et al., 2020)) was introduced into the best methylchavicol producer strain, SKF100_001079, or the best methyleugenol producer strain, SKF100_001080, to create SKF100_001119 and SKF100_001120, respectively. Enzyme abbreviations: FjTAL (*Flavobacterium johnsoniae* tyrosine ammonia lyase); SeC3H (*Saccharothrix espanensis* coumarate 3-hydroxylase); PkCOMT (*Populus kitakamiensis* caffeate 3-*O*-methyltransferase). **B** Methylchavicol (orange) and chavicol (red) titres produced by SKF100_001119 with and without supplementation of 3 mM l-tyrosine. **C** Methyleugenol (yellow), eugenol (blue), methylchavicol (orange) and chavicol (red) titres produced by SKF100_001120 with and without the supplementation of 3 mM l-tyrosine. All measurements were taken 24 h after induction and addition of the appropriate substrates. All experiments were performed using biological triplicates and error bars are representative of the standard deviations of these triplicates. Strains were grown in the absence (U) and presence (I) of IPTG.

In this study, we transformed the plasmid used for *p-*coumaric acid production (SBC007589 (Robinson et al., 2020)) into the previously described best methylchavicol producing strain SKF100_001079 to test the production of methylchavicol directly from l-tyrosine via a six enzymatic step pathway (Fig. 4A). The resulting strain (SKF100_001119) was grown in rich medium under both induced and uninduced conditions as well as with and without the supplementation of 3 mM l-tyrosine. Methylchavicol was detected even in the absence of supplemented l-tyrosine, and titres of up to 0.20 mM (29.6 mg L^−1^) were achieved in cultures of strain SKF100_001119 (Fig. 4B). It is worth noting that the higher methylchavicol titres observed when produced from l-tyrosine substrate compared to when produced from *p-*coumaric acid could be attributed to the higher observed cell density at the end of the 24 h production assay (Supplementary Fig. S2). This further points to the inhibition of *E. coli* growth in the presence of high concentrations of *p-*coumaric acid (Barthelmebs et al., 2001).

Similarly, to produce methyleugenol from l-tyrosine, we transformed the plasmid developed for *trans-*ferulic acid production (SBC010695 (Dunstan et al., 2020)) into the previously described best methyleugenol producing strain, SKF100_001080, with the aim of producing methyleugenol directly from l-tyrosine via an eight step metabolic pathway (Fig. 4A). The resulting strain, SKF100_001120, was grown in rich medium under both induced and uninduced conditions as well as with and without the supplementation of 3 mM l-tyrosine. Methylchavicol and methyleugenol were produced in cultures of SKF100_001120, under both induced and uninduced conditions (Fig. 4C). The simultaneous production of methylchavicol and methyleugenol was expected, given that *p-*coumaric acid is an intermediate of the *trans-*ferulic acid biosynthesis pathway and the enzymes of the monolignol and phenylpropene biosynthesis pathways exhibit substrate promiscuity.

In both the presence and absence of IPTG, chavicol titres accounted for ∼50% of the total phenylpropenes produced. Furthermore, methylchavicol titres up to 0.23 mM (34.1 mg L^−1^) were recorded (Fig. 4C). In contrast, in the presence of IPTG, eugenol only comprised up to ∼23% of the total phenylpropenes produced, with methyleugenol titres up to 0.04 mM (7.13 mg L^−1^) (Fig. 4C). *p-*Coumaric acid could either be directly reduced into its aldehyde by the SrCAR enzyme, and sequentially converted onwards to methylchavicol, or converted into caffeic acid and subsequently ferulic acid through the SeC3H and PkCOMT enzymes before being directed towards methyleugenol production. The higher final titres of methylchavicol compared with methyleugenol could suggest a preferential metabolic flux diverging from *p*-coumaric acid towards methylchavicol. Alternatively, there might exist an additional bottleneck present in the pathway towards methyleugenol production as a result of the additional two enzymatic steps towards *trans*-ferulic acid from *p*-coumaric acid.

The methyleugenol production pathway also comprised two different SAM-dependent OMTs that catalyse the production of ferulic acid from caffeic acid and methyleugenol from eugenol, resulting in competition over the availability of SAM. Additionally, there were no observable differences in final target titres achieved between strains grown with or without additionally supplementing 3 mM l-tyrosine (Fig. 4B and C). This likely points to limited l-tyrosine uptake into the cells during the 24-h production assay. Further experiments growing these strains in minimal media or in the absence of any exogenous l-tyrosine would be required to confirm the production of the target compounds directly from central carbon metabolism.

### 3.5 Biosynthesis of isoeugenol from ferulic acid

**Fig. 5:**
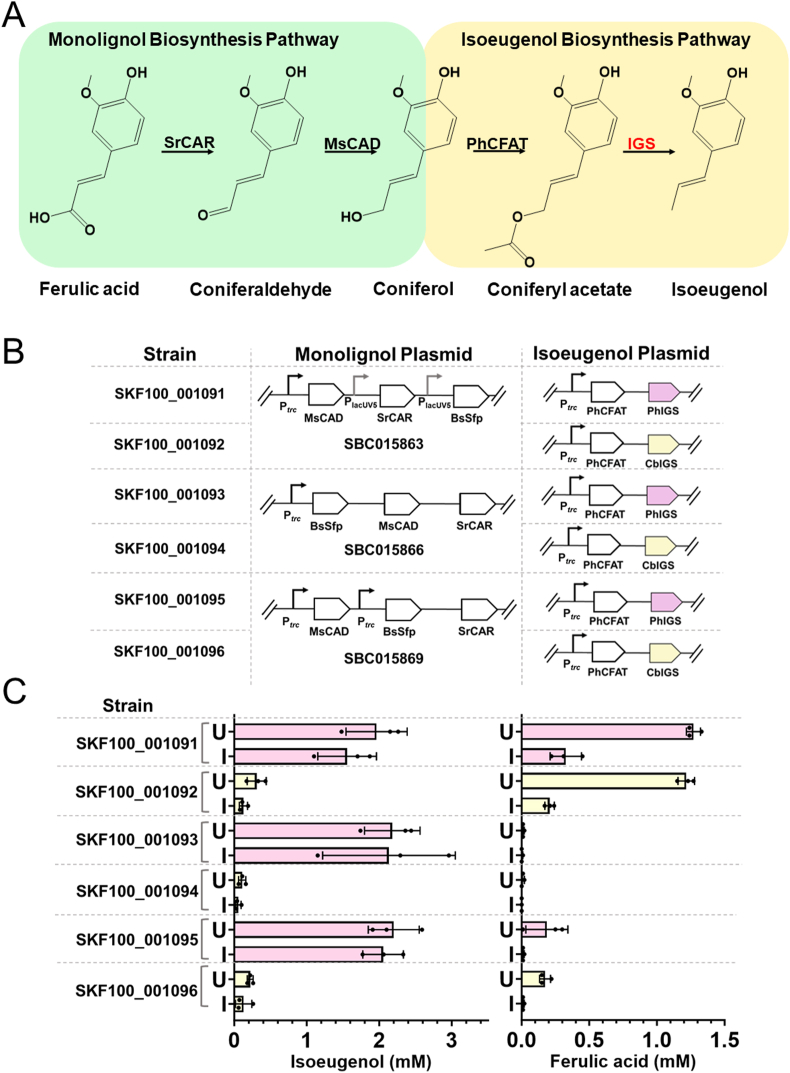
Microbial biosynthesis of isoeugenol by whole-cell bioconversion of *trans-*ferulic acid. **A** Monolignol (green) and isoeugenol (yellow) biosynthesis pathways. Enzyme abbreviations: SrCAR (*Segniliparus rugosus* carboxylic acid reductase); MsCAD (*Medicago sativa* cinnamyl alcohol dehydrogenase); PhCFAT (*Petunia hybrida* coniferyl alcohol acyltransferase); IGS (isoeugenol synthase). The monolignol biosynthesis plasmids (SBC015863, SBC015866 and SBC015869 (Hanko et al., 2023)) were used in this study. **B** Combination of plasmids co-transformed to generate the isoeugenol production strains used in this study (constructs not drawn to scale). **C** Library of strains producing isoeugenol (left bar chart) and concentration of unconverted *trans-*ferulic acid (right bar chart). Measurements taken from strains expressing PhIGS and CbIGS are shown in pink and yellow, respectively. All experiments were performed using biological triplicates and error bars are representative of the standard deviations of these triplicates. Error bars are representative of standard deviations of biological triplicates. Strains were grown in the absence (U) and presence (I) of IPTG.

To broaden the diversity of phenylpropene targets in this study, we further attempted to produce isoeugenol from its phenylacrylic acid precursor, *trans-*ferulic acid. We aimed to utilise the CAR-dependent monolignol biosynthesis pathway that comprises SrCAR, BsSfp and MsCAD for the conversion of *trans-*ferulic acid to coniferol and tested three different plasmid variations that have been shown to produce the highest coniferol titres (SBC015863, SBC015866, SBC015869) (Hanko et al., 2023). As previously described, the production of isoeugenol from coniferol has been accomplished in *E. coli* via two enzymatic steps (Kim et al., 2014). We planned to emulate these results by replacing ObEGS1 in plasmid SBC009876 with two isoeugenol synthase (IGS) enzyme candidates which should enable the production of isoeugenol instead of eugenol from coniferyl acetate (Fig. 5A). These modified plasmids were co-transformed with the three selected monolignol plasmid variations (Hanko et al., 2023) to assemble the isoeugenol biosynthesis library. The resulting strains, SKF100_001091 to SKF100_001096, were grown in rich media for 24 h in both induced and uninduced conditions after supplementation with 3 mM *trans-*ferulic acid (Fig. 5B).

In both induced and uninduced conditions, isoeugenol was detected in all strains (Fig. 5C). Although both enzyme candidates showed similar levels of substrate affinity to coniferyl acetate *in vitro* (Koeduka et al., 2008), PhIGS1, present in strains SKF100_001091, SKF100_001093 and SKF100_001095, outperformed CbIGS1 in strains SKF100_001092, SKF100_001094 and SKF100_001096. Large amounts of unconverted *trans-*ferulic acid were detected in SKF100_001091 and SKF100_001092, highlighting an inefficient reduction of *trans-*ferulic acid to its aldehyde form. These observations are in line with the original study (Hanko et al., 2023) whereby the plasmid SBC015863 (used in SKF100_001091 and SKF100_001092) only converted around 75% of the fed substrate. The best-performing strain, SKF100_001095, yielded titres of 2.20 mM (361.2 mg L^−1^) isoeugenol from 3 mM *trans-*ferulic acid, leaving behind only trace amounts of unconverted substrate. The high conversion rate and isoeugenol titres show potential for the extension of this pathway to produce methylisoeugenol in future work.

## 4 Conclusion

This study demonstrates the microbial production of methylchavicol and methyleugenol, as well as the production of isoeugenol from their phenylacrylic acid substrates. Furthermore, as l-tyrosine is naturally synthesised in *E. coli* through the shikimate pathway, production could be produced from central metabolism via upregulated tyrosine production rather than as a biotransformation from tyrosine supplied in the growth media. Although we were able to produce methyleugenol from l-tyrosine, the promiscuity of the enzymes used in this study works against its targeted production. Therefore, to achieve targeted production of methyleugenol from l-tyrosine, a CAR with higher affinity toward *trans-*ferulic acid and lower affinity toward *p-*coumaric acid might be required.

Additionally, future work could be directed toward the engineering of the host strain to enable the recycling of SAH back to SAM to increase the methylation efficiency of the OMT enzyme. This, coupled with the consolidation of the three biosynthesis pathways from l-tyrosine to phenylpropenes into a single plasmid, chromosomal integration of these genes into the host strain, or a combination of the two, could help realise the industrial potential of microbially-produced phenylpropenes.

## Funding

This project has received funding from the European Union's Horizon 2020 research and innovation programme under grant agreement 814408 SHIKIFACTORY100 – Modular cell factories for the production of 100 compounds from the shikimate pathway. The work of JeCh, ET and RB was supported through the Signals in the Soil program via UK Research and Innovation (UKRI; NE/T010959/1).

## CRediT authorship contribution statement

**Jeremy Chua:** Conceptualization, Formal analysis, Investigation, Visualization, Writing – original draft. **Erik K.R. Hanko:** Conceptualization, Writing – review & editing. **Andrew Yiakoumetti:** Investigation, Writing – review & editing. **Ruth A. Stoney:** Data curation, Writing – review & editing. **Jakub Chromy:** Data curation, Writing – review & editing. **Kris Niño G. Valdehuesa:** Data curation. **Katherine A. Hollywood:** Methodology. **Cunyu Yan:** Methodology. **Eriko Takano:** Funding acquisition, Supervision, Writing – review & editing. **Rainer Breitling:** Conceptualization, Funding acquisition, Project administration, Supervision, Writing – review & editing.

## Declaration of competing interest

The authors declare that they have no known competing financial interests or personal relationships that could have appeared to influence the work reported in this paper.

## Data Availability

Data will be made available on request.

## References

[bib1] Barthelmebs L., Diviès C., Cavin J.-F. (2001). Expression in Escherichia coli of native and chimeric phenolic acid decarboxylases with modified enzymatic activities and method for screening recombinant E. coli strains expressing these enzymes. Appl. Environ. Microbiol..

[bib2] Biała W., Jasiński M. (2018). The phenylpropanoid case–it is transport that matters. Front. Plant Sci..

[bib3] Brooks S.M. (2023). A tripartite microbial co-culture system for de novo biosynthesis of diverse plant phenylpropanoids. Nat. Commun..

[bib4] Carbonell P. (2018). Selenzyme: enzyme selection tool for pathway design. Bioinformatics.

[bib5] Chaieb K. (2007). The chemical composition and biological activity of clove essential oil, Eugenia caryophyllata (Syzigium aromaticum L. Myrtaceae): a short review. Phytother Res..

[bib6] Dunstan M.S. (2020). Engineering Escherichia coli towards de novo production of gatekeeper (2S)-flavanones: naringenin, pinocembrin, eriodictyol and homoeriodictyol. Synth. Biol..

[bib7] Gang D.R. (2001). An investigation of the storage and biosynthesis of phenylpropenes in sweet basil. Plant Physiol..

[bib8] Gang D.R. (2002). Characterization of phenylpropene O-methyltransferases from sweet basil: facile change of substrate specificity and convergent evolution within a plant O-methyltransferase family. Plant Cell.

[bib9] Hahlbrock K., Scheel D. (1989). Physiology and molecular biology of phenylpropanoid metabolism. Annu. Rev. Plant Biol..

[bib10] Han D. (2013). Bacterial biotransformation of phenylpropanoid compounds for producing flavor and fragrance compounds. J. Korean Soc. Appl. Biol. Chem..

[bib11] Hanko E.K.R. (2023). Carboxylic acid reductase-dependent biosynthesis of eugenol and related allylphenols. PREPRINT (Version 1) available at Research Square.

[bib12] Humphreys J.M., Chapple C. (2002). Rewriting the lignin roadmap. Curr. Opin. Plant Biol..

[bib13] Ismaiel O.A. (2016). Determination of estragole in pharmaceutical products, herbal teas and herbal extracts using GC-FID. J. Appl. Pharmaceut. Sci..

[bib14] Jirovetz L. (2006). Chemical composition and antioxidant properties of clove leaf essential oil. J. Agric. Food Chem..

[bib15] Kim S.-J. (2014). Allyl/propenyl phenol synthases from the creosote bush and engineering production of specialty/commodity chemicals, eugenol/isoeugenol, in Escherichia coli. Arch. Biochem. Biophys..

[bib16] Koeduka T. (2014). The phenylpropene synthase pathway and its applications in the engineering of volatile phenylpropanoids in plants. Plant Biotechnol..

[bib17] Koeduka T. (2018). Functional evolution of biosynthetic enzymes that produce plant volatiles. Biosci., Biotechnol., Biochem..

[bib18] Koeduka T. (2008). The multiple phenylpropene synthases in both Clarkia breweri and Petunia hybrida represent two distinct protein lineages. Plant J..

[bib19] Koeduka T. (2009). Biosynthesis of t-anethole in anise: characterization of t-anol/isoeugenol synthase and an O-methyltransferase specific for a C7-C8 propenyl side chain. Plant Physiol..

[bib20] Kunjapur A.M., Hyun J.C., Prather K.L. (2016). Deregulation of S-adenosylmethionine biosynthesis and regeneration improves methylation in the E. coli de novo vanillin biosynthesis pathway. Microb. Cell Factories.

[bib21] Lee H.L. (2019). Synthesis of methylated anthranilate derivatives using engineered strains of Escherichia coli. J. Microbiol. Biotechnol..

[bib22] Li K., Frost J. (1998). Synthesis of vanillin from glucose. J. Am. Chem. Soc..

[bib23] Liu Q., Lin B., Tao Y. (2022). Improved methylation in E. coli via an efficient methyl supply system driven by betaine. Metab. Eng..

[bib24] Loenen W. (2006). S-adenosylmethionine: jack of all trades and master of everything?. Biochem. Soc. Trans..

[bib25] Program N.T. (2000). NTP toxicology and carcinogenesis studies of methyleugenol (CAS NO. 93-15-2) in F344/N rats and B6C3F1 mice (gavage studies). Natl. Toxicol. Progr. Tech. Rep..

[bib26] Robinson C.J. (2020). Rapid prototyping of microbial production strains for the biomanufacture of potential materials monomers. Metab. Eng..

[bib27] Roe A.J. (2002). *Inhibition of* Escherichia coli *growth by acetic acid: a problem with methionine biosynthesis and homocysteine toxicity*. Microbiology (Read.).

[bib28] Rosano G.L., Ceccarelli E.A. (2014). Recombinant protein expression in Escherichia coli: advances and challenges. Front. Microbiol..

[bib29] Swainston N. (2018). PartsGenie: an integrated tool for optimizing and sharing synthetic biology parts. Bioinformatics.

[bib30] Tegel H., Ottosson J., Hober S. (2011). Enhancing the protein production levels in Escherichia coli with a strong promoter. FEBS J..

[bib31] Vassão D.G. (2007). A pinoresinol–lariciresinol reductase homologue from the creosote bush (Larrea tridentata) catalyzes the efficient in vitro conversion of p-coumaryl/coniferyl alcohol esters into the allylphenols chavicol/eugenol, but not the propenylphenols p-anol/isoeugenol. Arch. Biochem. Biophys..

[bib32] Wang J. (1997). Floral scent production in Clarkia breweri (Onagraceae). II. Localization and developmental modulation of the enzyme S-adenosyl-L-methionine:(iso)eugenol O-methyltransferase and phenylpropanoid emission. Plant Physiol..

[bib33] Wang J., Pichersky E. (1998). Characterization of S-Adenosyl-l-methionine:(Iso) eugenol O-methyltransferase involved in floral scent production in Clarkia breweri. Arch. Biochem. Biophys..

[bib34] Wei P.-P. (2021). Engineering a heterologous synthetic pathway in Escherichia coli for efficient production of biotin. Biotechnol. Lett..

[bib35] Wu S. (2003). Two O-methyltransferases isolated from flower petals of Rosa chinensis var. spontanea involved in scent biosynthesis. J. Biosci. Bioeng..

[bib36] Yahyaa M. (2019). Biosynthesis of methyleugenol and methylisoeugenol in Daucus carota leaves: characterization of eugenol/isoeugenol synthase and O-Methyltransferase. Phytochemistry.

[bib37] Yauk Y.K. (2015). The O-methyltransferase gene MdoOMT1 is required for biosynthesis of methylated phenylpropenes in ripe apple fruit. Plant J..

[bib38] Zhang J., Zheng Y.G. (2016). SAM/SAH analogs as versatile tools for SAM-dependent methyltransferases. ACS Chem. Biol..

[bib39] Zhang C., Sultan S.A., Chen X. (2021). Biotechnological applications of S-adenosyl-methionine-dependent methyltransferases for natural products biosynthesis and diversification. Bioresour. Bioproc..

