# Supplementary material for: Bioproduction of methylated phenylpropenes and isoeugenol in Escherichia coli
Source: Metab Eng Commun. 2024 May 15;18:e00237. doi: 10.1016/j.mec.2024.e00237 (PMC11127157; doi:10.1016/j.mec.2024.e00237)
Supplement: Multimedia component 1 [file mmc1.docx]

**Supporting Information**

**Biosynthesis of methylated phenylpropenes and isoeugenol in *Escherichia coli***

Jeremy Chua^1^, Erik K. R. Hanko^1^, Andrew Yiakoumetti^1^, Ruth A. Stoney^1^, Jakub Chromy^1^, Kris Niño G. Valdehuesa^1^, Katherine A. Hollywood^1^, Cunyu Yan^1^, Eriko Takano^1^, Rainer Breitling^1^

^1^Manchester Institute of Biotechnology, Faculty of Science and Engineering, University of Manchester, 131 Princess Street, Manchester M1 7DN, United Kingdom

Corresponding author:

Rainer Breitling
rainer.breitling@manchester.ac.uk

# Supplementary Methods

## Plasmid construction

**SBC015898** was constructed by HiFi DNA Assembly. Oligonucleotide primers EH184_f and EH185_r were used to amplify EOMT1 from SBC013268. The PCR product was combined with BamHI-digested SBC009876.

**SBC015899** was constructed by HiFi DNA Assembly. Oligonucleotide primers EH188_f and EH185_r were used to amplify EOMT1 from SBC013268. Oligonucleotide primers EH186_f and EH187_r were used to amplify the intergenic region, comprising a *trc* promoter, from SBC006456. The PCR products were combined with BamHI-digested SBC009876.

**SBC015900** was constructed by HiFi DNA Assembly. Oligonucleotide primers EH189_f and EH190_r were used to amplify IEMT1 from SBC013270. The PCR product was combined with BamHI-digested SBC009876.

**SBC015901** was constructed by HiFi DNA Assembly. Oligonucleotide primers EH191_f and EH190_r were used to amplify IEMT1 from SBC013270. Oligonucleotide primers EH186_f and EH187_r were used to amplify the intergenic region, comprising a *trc* promoter, from SBC006456. The PCR products were combined with BamHI-digested SBC009876.

**SBC015902** was constructed by HiFi DNA Assembly. Oligonucleotide primers EH192_f and EH193_r were used to amplify RcOMT1 from SBC013272. The PCR product was combined with BamHI-digested SBC009876.

**SBC015903** was constructed by HiFi DNA Assembly. Oligonucleotide primers EH194_f and EH193_r were used to amplify RcOMT1 from SBC013272. Oligonucleotide primers EH186_f and EH187_r were used to amplify the intergenic region, comprising a *trc* promoter, from SBC006456. The PCR products were combined with BamHI-digested SBC009876.

**SBC015904** was constructed by HiFi DNA Assembly. Oligonucleotide primers EH195_f and EH196_r were used to amplify AIMT1 from SBC013372. The PCR product was combined with BamHI-digested SBC009876.

**SBC015905** was constructed by HiFi DNA Assembly. Oligonucleotide primers EH197_f and EH196_r were used to amplify AIMT1 from SBC013372. Oligonucleotide primers EH186_f and EH187_r were used to amplify the intergenic region, comprising a *trc* promoter, from SBC006456. The PCR products were combined with BamHI-digested SBC009876.

**SBC015906** was constructed by HiFi DNA Assembly. Oligonucleotide primers EH198_f and EH199_r were used to amplify MdoOMT1aΔ from SBC013374. The PCR product was combined with BamHI-digested SBC009876.

**SBC015907** was constructed by HiFi DNA Assembly. Oligonucleotide primers EH200_f and EH199_r were used to amplify MdoOMT1aΔ from SBC013374. Oligonucleotide primers EH186_f and EH187_r were used to amplify the intergenic region, comprising a *trc* promoter, from SBC006456. The PCR products were combined with BamHI-digested SBC009876.

**SBC015908** was constructed by HiFi DNA Assembly. Oligonucleotide primers EH201_f and EH202_r were used to amplify MdoOMT1b from SBC013376. The PCR product was combined with BamHI-digested SBC009876.

**SBC015909** was constructed by HiFi DNA Assembly. Oligonucleotide primers EH203_f and EH202_r were used to amplify MdoOMT1b from SBC013376. Oligonucleotide primers EH186_f and EH187_r were used to amplify the intergenic region, comprising a *trc* promoter, from SBC006456. The PCR products were combined with BamHI-digested SBC009876.

**SBC015910** was constructed by HiFi DNA Assembly. Oligonucleotide primers EH204_f and EH205_r were used to amplify PhCFAT from SBC009876. Oligonucleotide primers EH206_f and EH207_r were used to amplify PhIGS1 from SBC013264. The PCR products were combined with EcoRI/BamHI/HindIII-digested SBC009876.

**SBC015911** was constructed by HiFi DNA Assembly. Oligonucleotide primers EH204_f and EH205_r were used to amplify PhCFAT from SBC009876. Oligonucleotide primers EH208_f and EH209_r were used to amplify CbIGS1 from SBC013266. The PCR products were combined with EcoRI/BamHI/HindIII-digested SBC009876.

## GC-MS analysis

Phenylpropene targets were detected and analysed using an Agilent Technologies 5975 Series MSD mass spectrometer coupled to a 7890B GC and a 7693 autosampler. A VF-5HT column (30 m x 250 µm x 0.1 µm, Agilent Technologies) was used for separation. The MS was equipped with an electron impact ion source using 70 eV ionisation and a fixed emission of 35 µA. Mass spectra were collected for the range of 50–550 m/z at a frequency of 5.5 scans/s and a cycle time of 181.35 ms.

The samples were analysed with an inlet temperature of 240°C and a split ratio of 30:1. Helium was used as the carrier gas with a flow rate of 1 mL/min and a pressure of 7.6 psi. The chromatography was programmed to begin at 50°C with a hold time of 0.5 min, followed by an increase to 170°C at a rate of 120°C/min, a subsequent increase to 280°C at a rate of 70°C/min, and a final increase to 350°C at a rate of 120°C/min. The temperature was held at 350°C for 1 min. The injected sample volume was 1 µL and the total runtime per analysis was 4.655 min. Sec-B had a retention time of 2.27 min and a mass-to-charge ratio of 105.1 m/z. Eugenol had a retention time of 2.88 min and a mass-to-charge ratio of 164.2 m/z. Methyleugenol had a retention time of 2.95 min and a mass-to-charge ratio of 178.2 m/z. Chavicol had a retention time of 2.71 min and a mass-to-charge ratio of 134.2 m/z. Methylchavicol had a retention time of 2.61 min and a mass-to-charge ratio of 148.2 m/z. Isoeugenol had a retention time of 3.05 min and a mass-to-charge ratio of 164.2 m/z (Supplementary Figure S3A).

## UPLC-DAD analysis

Analysis was conducted using an Agilent 1290 Series UPLC with diode array detection. Phenylacrylic acid targets (ferulic acid and coumaric acid) were analysed using a Waters Acquity BEH C18 column (50 mm x 2.1 mm x 1.7 µm) at 45°C with a flow rate of 0.6 mL/min. An optimum separation gradient was obtained with a binary mobile phase A (H_2_O, 0.1% formic acid [FA]) and B (MeOH, 0.1% FA). The gradient elution program was: 0–2.3 min, 75–5% A; 2.3–3 min, 5–75% A; 3–3.3 min, hold at 75% A. The injected sample volume was 5 µL. Coumaric acid was measured with a wavelength of 310 nm with a retention time of 0.92 min. Ferulic acid was measured with a wavelength of 324 nm with a retention time of 1.11 min (Supplementary Figure S3B).

## LC-MS/MS analysis

Tyrosine was quantified using a Waters Xevo TQ-S equipped with a H Class UPLC and using a Waters Acquity HSS T3 column (50 mm x 2.1 mm x 1.8 µm). The desolvation gas flow rate was set to 1000 L/h at a temperature of 600°C. The cone gas flow rate was fixed at 0.15 mL/min and the source temperature at 150°C. The source offset was set to 50 V. The capillary voltage was optimised at 1.0 kV for positive (ESI^+^) mode. A dwell time of 33 ms/transition was chosen. The column temperature was set at 45°C with a mobile phase flow rate of 0.6 mL/min. An optimum separation gradient was obtained with a binary mobile phase A (H_2_O, 0.1% FA) and B (MeOH, 0.1% FA). The gradient elution program was: 0–1 min, 99–19% A; 1–1.1 min, 19–5% A; 1.1–1.5 min, held at 5% A, 1.5–3 min, 5–99% A. The injected sample volume was 1 µL. Tyrosine was monitored in ESI^+^ mode with a precursor ion mass of m/z=182.06, product ion mass of 136.12 m/z, cone voltage of 20 V, collision energy of 13 eV and a retention time of 0.67 min (Supplementary Figure S3C).

# Supplementary Tables

**Supplementary Table S1.** Strain name, genotype and description of the strains constructed and used in this study.

| **Name** | **Genotype** | **Source** | **Description** |
| --- | --- | --- | --- |
| *Escherichia coli* NEB 5α | *fhuA2Δ(argF-lacZ)U169 phoA glnV44 Φ80Δ(lacZ)M15 gyrA96 recA1 relA1 endA1 thi-1 hsdR17* | NEB (C2987-8) | General cloning and pathway expression host |
| SKF100_001079 | NEB 5α SBC015869 SBC015898 | *This study* | Methylchavicol/methyleugenol producing strain |
| SKF100_001080 | NEB 5α SBC015869 SBC015899 | *This study* | Methylchavicol/methyleugenol producing strain |
| SKF100_001081 | NEB 5α SBC015869 SBC015900 | *This study* | Methyleugenol producing strain |
| SKF100_001082 | NEB 5α SBC015869 SBC015901 | *This study* | Methyleugenol producing strain |
| SKF100_001083 | NEB 5α SBC015869 SBC015902 | *This study* | Methylchavicol/methyleugenol producing strain |
| SKF100_001084 | NEB 5α SBC015869 SBC015903 | *This study* | Methylchavicol/methyleugenol producing strain |
| SKF100_001085 | NEB 5α SBC015869 SBC015904 | *This study* | Methylchavicol/methyleugenol producing strain |
| SKF100_001086 | NEB 5α SBC015869 SBC015905 | *This study* | Methylchavicol/methyleugenol producing strain |
| SKF100_001087 | NEB 5α SBC015869 SBC015906 | *This study* | Methylchavicol/methyleugenol producing strain |
| SKF100_001088 | NEB 5α SBC015869 SBC015907 | *This study* | Methylchavicol/methyleugenol producing strain |
| SKF100_001089 | NEB 5α SBC015869 SBC015908 | *This study* | Methylchavicol/methyleugenol producing strain |
| SKF100_001090 | NEB 5α SBC015869 SBC015909 | *This study* | Methylchavicol/methyleugenol producing strain |
| SKF100_001091 | NEB 5α SBC015863 SBC015910 | *This study* | Isoeugenol producing strain |
| SKF100_001092 | NEB 5α SBC015863 SBC015911 | *This study* | Isoeugenol producing strain |
| SKF100_001093 | NEB 5α SBC015866 SBC015910 | *This study* | Isoeugenol producing strain |
| SKF100_001094 | NEB 5α SBC015866 SBC015911 | *This study* | Isoeugenol producing strain |
| SKF100_001095 | NEB 5α SBC015869 SBC015910 | *This study* | Isoeugenol producing strain |
| SKF100_001096 | NEB 5α SBC015869 SBC015911 | *This study* | Isoeugenol producing strain |
| SKF100_001119 | NEB 5α SBC007589 SBC015869 SBC015898 | *This study* | Methylchavicol producing strain from tyrosine |
| SKF100_001120 | NEB 5α SBC010695 SBC015869 SBC015899 | *This study* | Methyleugenol producing strain from tyrosine |

**Supplementary Table S2.** Enzyme candidates used in this study.

| **Enzyme abbreviation** | **Enzyme activity** | **EC number** | **UniProt ID** | **Species source** | **Gene part ID** | **Reference** |
| --- | --- | --- | --- | --- | --- | --- |
| EOMT1 | SAM-dependent OMT | 2.1.1.146 | Q93WU2 | *Ocimum basilicum* | SBC013268 | [1] |
| IEMT1 | SAM-dependent OMT | 2.1.1.146 | O04385 | *Clarkia breweri* | SBC013270 | [2] |
| RcOMT1 | SAM-dependent OMT | 2.1.1.146 | Q7X9J1 | *Rosa chinensis* var. *spontanea* | SBC013272 | [3] |
| AIMT1 | SAM-dependent OMT | 2.1.1.279 | B8RCD3 | *Pimpinella anisum* | SBC013372 | [4] |
| MdoOMT1AΔ | SAM-dependent OMT | 2.1.1. | 27AA N-terminal truncated version of A0A0N7CLJ4 | *Malus domestica 'Gala'* | SBC013374 | [5] |
| MdoOMT1b | SAM-dependent OMT | 2.1.1. | A0A0N7CS03 | *Malus domestica 'Gala'* | SBC013376 |  |
| PhIGS1 | Isoeugenol synthase | 1.1.1.319 | Q15GI3 | *Petunia hybrida* | SBC013264 | [6] |
| CbIGS1 | Isoeugenol synthase | 1.1.1.319 | B2WSM8 | *Clarkia breweri* | SBC013266 |  |

**Supplementary Table S3.** Description, nucleotide sequence and application of the primers used in this study.

| **Primer name** | **Description** | **Primer sequence (5' to 3')** | **Application** |
| --- | --- | --- | --- |
| EH184_f | PCR amplify EOMT1 from SBC013268 for SBC015898 | ggctgcgttttgaggaaggaaaggacaatccccg | Hi-Fi Assembly |
| EH185_r | PCR amplify EOMT1 from SBC013268 for SBC015898/899 | gatccttactcgagtttggatccttatggataggcctcgataaggc | Hi-Fi Assembly |
| EH186_f | PCR amplify intergenic region from SBC006456 for SBC015899/901/903/905/907/909 | ggctgcgttttgaggataacaaacaagaagtaacttatcggcag | Hi-Fi Assembly |
| EH187_r | PCR amplify intergenic region from SBC006456 for SBC015899/901/903/905/907/909 | tcgcttccctacggcggt | Hi-Fi Assembly |
| EH188_f | PCR amplify EOMT1 from SBC013268 for SBC015899 | cgtagggaagcgaaggaaaggacaatccccg | Hi-Fi Assembly |
| EH189_f | PCR amplify IEMT1 from SBC013270 for SBC015900 | ggctgcgttttgaggagtaacaaatgtaattcagcttgttagcg | Hi-Fi Assembly |
| EH190_r | PCR amplify IEMT1 from SBC013270 for SBC015900/901 | gatccttactcgagtttggatccttaagccgttttcagaaactccatc | Hi-Fi Assembly |
| EH191_f | PCR amplify IEMT1 from SBC013270 for SBC015901 | cgtagggaagcgagtaacaaatgtaattcagcttgttagcg | Hi-Fi Assembly |
| EH192_f | PCR amplify RcOMT1 from SBC013272 for SBC015902 | ggctgcgttttgaggacaattatatgaacaaagcccgacg | Hi-Fi Assembly |
| EH193_r | PCR amplify RcOMT1 from SBC013272 for SBC015902/903 | gatccttactcgagtttggatcctcacttgatgaactccatcacc | Hi-Fi Assembly |
| EH194_f | PCR amplify RcOMT1 from SBC013272 for SBC015903 | cgtagggaagcgacaattatatgaacaaagcccgacg | Hi-Fi Assembly |
| EH195_f | PCR amplify AIMT1 from SBC013372 for SBC015904 | ggctgcgttttgaggaaccaactatgagctttacccg | Hi-Fi Assembly |
| EH196_r | PCR amplify AIMT1 from SBC013372 for SBC015904/905 | gatccttactcgagtttggatccttatttctttttataaagttcgaccacccc | Hi-Fi Assembly |
| EH197_f | PCR amplify AIMT1 from SBC013372 for SBC015905 | cgtagggaagcgaaccaactatgagctttacccg | Hi-Fi Assembly |
| EH198_f | PCR amplify MdoOMT1aΔ from SBC013374 for SBC015906 | ggctgcgttttgaggagattcgctatccctacctcc | Hi-Fi Assembly |
| EH199_r | PCR amplify MdoOMT1aΔ from SBC013374 for SBC015906/907 | gatccttactcgagtttggatccttacggatagacttcaatcaagctg | Hi-Fi Assembly |
| EH200_f | PCR amplify MdoOMT1aΔ from SBC013374 for SBC015907 | cgtagggaagcgagattcgctatccctacctcc | Hi-Fi Assembly |
| EH201_f | PCR amplify MdoOMT1b from SBC013376 for SBC015908 | ggctgcgttttgaggatggcctgagcgaggctca | Hi-Fi Assembly |
| EH202_r | PCR amplify MdoOMT1b from SBC013376 for SBC015908/909 | gatccttactcgagtttggatcctcatgggtacacttcaattaaagagcg | Hi-Fi Assembly |
| EH203_f | PCR amplify MdoOMT1b from SBC013376 for SBC015909 | cgtagggaagcgatggcctgagcgaggctca | Hi-Fi Assembly |
| EH204_f | PCR amplify PhCFAT from SBC009876 for SBC015910/911 | gaattgtgagcggataacaatttcag | Hi-Fi Assembly |
| EH205_r | PCR amplify PhCFAT from SBC009876 for SBC015910/911 | ttagtacgttgcggtcaaatctag | Hi-Fi Assembly |
| EH206_f | PCR amplify PhIGS1 from SBC013264 for SBC015910 | accgcaacgtactaaagtgctttgggtctactgg | Hi-Fi Assembly |
| EH207_r | PCR amplify PhIGS1 from SBC013264 for SBC015910 | gatccttactcgagtttggatccttatgtcgacggctgcgc | Hi-Fi Assembly |
| EH208_f | PCR amplify CbIGS1 from SBC013266 for SBC015911 | accgcaacgtactaaagctgcgtttcctcgaaa | Hi-Fi Assembly |
| EH209_r | PCR amplify CbIGS1 from SBC013266 for SBC015911 | gatccttactcgagtttggatcctcattcaaattcggccagg | Hi-Fi Assembly |

**Supplementary Table S4.** Sequences of synthesised DNA fragments. Gene coding sequences are highlighted in uppercase. Restriction sites, EcoRI and BamHI, are underlined.

| **Enzyme** | **Sequence** |
| --- | --- |
| ObEOMT1 | gaattcaaaagatctgagtcttgtacccgttggccgcgggaggaaaggacaatccccgccaacggattagtccttctattatagtcgcagggggtaaagcATGGCACTCCAAAAAGTGGATATCTCATTGAGCACGGAACAGCTGTTACAAGCCCAAGTTCATGTTTGGAACCACATGTACGCCTTTGCCAACAGCATGTCGTTAAAGTGCGCGATACAGCTGGGCATTCCAGACATTTTACATAAACATGGCCGACCTATGACACTGTCTCAGCTGTTGCAGTCAATTCCTATTAATAAAGAGAAAACCCAATGTTTTCAGAGGCTGATGCGTGCGTTAGTGAACTCAAACTTCTTCATTGAAGAAAATAATAGTAATAATCAAGAAGTGTGCTATTGGCTCACTCCGGCGAGCTGTCTGCTGCTGAAGGAAGCACCCCTGACCGTCACGCCCCTGGTGCAGGTTGTGTTAGACCCGACTTTTACAAACCCGTGGCATCACATGAGCGAATGGTTTACGCACGAAAAACACGCGACCCAATTTGAAGCCGCAAATGGGTGTACGTTCTGGGAAAAGTTAGCGAATGAGCCAAGTAAAGGACGCTTCTTCGATGAAGCCATGAGCTGCGATAGTCGTCTAATTGCACATGTGTTCACGAAAGACTACAAACACGTGATCGAGGGCATTCGCACGCTCGTAGACGTGGGCGGCGGCAACGGCACAATGGCAAAGGCCATTGTCGAAGCGATGCCCACCATTAAGTGTACCGTGATTGACTTACCGCATGTTGTGGCGGGTCTGGAAAGTACCGATAACTTGAACTATATAGGCGGTGATATGTTTCAATCTATTCCTAGCGCGGATGCGATCCTGCTTAAGAGCATCATCCACGATTGGGATGACGTGGAGGGCCTTAAAATTCTGAAGAAGTGTAAGGACGCGGTGGTAATGGGCGGTAAAGTAATAATCATCGATGTAGTTGTCGGCGTGAATCACGATATCGACGAGGTGCTTGAAGACCAGCTACATTTCGATATGGCGATGATGTGCTACTTTAACGCGAAAGAGCGCACTATGTCCGAATGGGAAAAATTGATCTACGATGCAGGCTTCAAAAGTTATAAGCTGACCCCAGCCTTTGGCGTTCGCAGCCTTATCGAGGCCTATCCATAAttgtagactcggatccaaactcgag |
| CbIEMT1 | gaattcaaaagatctgagtcttgtagatgacacctctaagagaacgcgcggtaacaaatgtaattcagcttgttagcggtcacgctgaccgcctctgaagggaggtaggaATGGGCTCAACCGGCAACGCGGAAATCCAGATTATTCCGACGCATAGCAGCGACGAAGAGGCCAATCTTTTCGCAATGCAGCTCGCTTCCGCGGCTGTCCTGCCGATGGCCCTGAAAGCGGCTATTGAGTTGGATGTTTTGGAGATCATGGCAAAAAGTGTACCGCCGAGCGGGTACATCTCGCCCGCTGAAATTGCCGCACAGCTGCCGACGACTAATCCAGAAGCGCCAGTGATGCTGGACCGTGTCCTGCGCCTGCTGGCATCTTACTCTGTAGTTACTTACACCTTACGCGAGCTGCCGAGCGGTAAGGTTGAACGTCTCTATGGCTTGGCACCTGTGTGTAAATTTTTGACCAAAAATGAGGATGGCGTGTCGCTCGCACCGTTCCTCCTGACGGCGACGGATAAAGTACTATTAGAACCGTGGTTCTACCTAAAAGATGCCATTTTAGAGGGCGGGATTCCGTTCAACAAAGCTTACGGCATGAACGAATTTGATTACCATGGCACAGATCATCGATTTAACAAAGTTTTTAACAAGGGAATGTCGAGTAATTCGACAATAACCATGAAGAAGATATTGGAAATGTACAACGGGTTTGAAGGCCTTACAACGATTGTGGATGTAGGCGGAGGCACTGGTGCGGTAGCCAGCATGATTGTTGCGAAGTATCCTAGCATCAATGCTATTAATTTTGATCTGCCGCATGTGATCCAGGACGCACCCGCCTTTAGCGGCGTGGAACATCTGGGCGGCGATATGTTCGACGGAGTGCCTAAAGGCGATGCTATTTTCATTAAATGGATTTGTCATGACTGGAGCGACGAACATTGCCTCAAACTTCTGAAAAACTGTTACGCTGCCCTCCCGGATCATGGGAAAGTTATCGTTGCAGAATACATTCTACCGCCCAGTCCTGATCCAAGCATTGCAACCAAGGTAGTCATTCACACAGATGCACTAATGCTAGCATACAATCCGGGAGGAAAGGAACGCACCGAGAAGGAATTTCAGGCCTTAGCCATGGCGAGCGGTTTCCGTGGTTTCAAAGTGGCGTCCTGCGCATTTAATACTTATGTGATGGAGTTTCTGAAAACGGCTTAAttgtagactcggatccaaactcgag |
| RcOMT1 | gaattcaaaagatctgagtcttgtaagaacctgtctataaaccaatacattgttaaacagttaatcaggcaattatatgaacaaagcccgacgactactatcaggggagtattgagaaggaggcattgtATGGCCAGCCCGTTAGAAATTGAAATCTTCGGCTGCCCATCTATCCATGTAGATGCGAAACGCAAAGAAGAAGAAAGTTCGTTTCATCATGCGGTGCAGCTGATGCTGTCTTCCGTGCTGCCGATGAGTATGCAATTGGCTATCGACCTGGGACTGTTCGATGTGATTGCCAAAGCAGGCACCGATGCCAAGCTTTCCGCCCTTGATATCGCGGCCAAGATTGGTACCAAAAACCCGCATGCTCCGGTGACCCTGGATCGCATTCTTCGGTTATTGACCGCTCATTCTGTACTAAGCTGCAGCGTGGTAACAGGGCAGCGTCTGTACAGTCTGACGGCGGTTTCGAAGCACTTTGTAACCAGCGAAGATGGCGCGTCGCTCTCACCGGTGATGGCGTCTATCCAAGCAAACGTTGTGATGAACTCTTGGTCGCAGGTTAAAGATGCCATTGTGGAAGGTGGCATCCCGTTCAACCGCGTCCATGGCAAACACTTCTTCGAATATGCAGATTCGGACCCTCGCTTCAACCAGGTGTTCAACTCGGGAATGGTGAATCTGACCACCCTGGTGATGCGCCGCATCCTTGATAGTTATCAAGGTTTTGAACATCTCACCCAGGTTGTGGATGTCGGCGGCGGCCTGGGAGTTGCCCTAGGTTTAATTACCAGTCGTTATCCACATATCAAGGGCGTGAATTACGATCTTCCACATGTTATTAAGCATGCGCCGCATTACCCAGGCGTACAGCATGTTGGCGGTGATATGTTCTCCAACGTTCCGAGTGGTGATGCGATTTTTATGAAAAATATTCTGCATGACTGGATGGATGAACAGTGCATTAAATTGCTTAAAAACTGGTATACGGCAATCCCAGATAACGGTAAAGTTATCGTCGTGGAAGCACTTGTCTCAGTTGAACCGGACACCTCTCCTGCAGAAAAGATCACCTCGGACTTCGACGTGCTGATGATGACACTGTCGCCTGGGGGTAAAGAACGTACCCAGCACGAATTTATGGACTTAGCTAATGCAGCAGGCTTTTCCGCAATCAAATATGAGTGCTTAAGTTCATACCTGCGGGTGATGGAGTTCATCAAGTGAttgtagactcggatccaaactcgag |
| PaAIMT1 | gaattcaaaagatctgagtcttgtatgacttaacaacaaccataaggagcactgcaccaactatgagctttacccgtaatgcgtttttctatggacaagagggggtaatacaaacATGGCAAGCCACGATCAAGAGGCGTTTCTGACCGCCATGCAAATCGTGAACAGCAGCGCCGTCGACGGTGTGCTCATCTGTCTCATCGAACTGAATGTGTTTGACATTATGATGCAGAAAGCTGGAATGGATGGCTACCTGCATCCGGATGAAATTGCATTAAATCTGCCGACCAAAAATCCGCAAGCGCCGGAAATGCTGGACCGTATGTTGCGTATTCTGGCGTCGCATAGCATCATTAAGTGCAAGTTAGTCAAAAAGATGTCCGGAAATGCCCTGCTTACCCGCGCCTATGGTCTGACCTTAATTAGTCAGTACTTTGTTAACGCTCAAGATGGCCCATGTCTTGCGCCGTACCTGAAATTGATCCATCATAAACAGATGCAAAATTCATGGGAAAAAGTAAATGAGGCCGTGCTAGAAGGTGGATACGCGTTTAACAAAGCCCATGCAGGCTCTACCTTCTTTGAATACCTGGGCAAGGATAAGTCGGTAGCGGAGTTACTGTCTCAAACCATGGCCAAATCGATTCCGACCAGTATGAATATTCTGCTGAAAAGCTATAAAGGCTTCGAAGGTGTGAAAGAAGTGGTTGATGTTGGGGGCGCCTATGCGGCAACCCTGAGCTGCATTATTAGTTTCAACCCGCACGTAAAAGGTATCAATTTCGATGTGCCTCACGTGATCAAAAACGCGCCGAGCCTGCCGGGTATTACGCATGTTGGGGGCGACATGTTTGAAAGCGTCCCGCGCGGCGAGGCCATCGTTTTACAGCGCGTCCTGCACGATTGGACTGACGAAGAGAGCGTTAAAATCTTAAAGAAATGTTATGAGGCGATTCCGGATCATGGTAAAGTTGTGATTATTGAGATGATACAGACAGAAATGCCTGAGGATGACATTATCGCGAAAAACATCAGTGAAATGGATATCCGCATGTTACTGTACACACCTGGCGGTAAAGAACGGACCGTTAACGAGTTCCTGATGTTGGGCAAGCAGGCCGGATTCCCGTCCTCAAAGTACATTTGTGGTGCGGACTTGTATGGGGTGGTCGAACTTTATAAAAAGAAATAAttgtagactcggatccaaactcgag |
| MdoOMT1AΔ | gaattcaaaagatctgagtcttgtatggacgttagcgtccagttcaatcatgagattcgctatccctacctccatgctcgataactgactgtaagataggaggaggtccgaatATGTCACTGAGCAATGAAGTGGGTGCAACCAGTCACGAGCTTCTGGGGGCCCAAGCGCAACTGTGGAATCACATTTTCCAGTTCATAAACTCAATGTCACTTAAATGCGCCGTTCAGCTGGGCATTGCCGACGTCATCCACAATCATGGTCAGCCGATTTCTCTCAGTGAGTTAATGGCCGGCCTCAAAGTGCACCCGTCTAAGGCGCATTTTGTGTCTCGTCTGATGCGCATTCTGGTGCATAGTAACTTCTTTGCCCAACATCACCACGTACATCAGGATCGAGCGGATGTCGAAGAAGAAGAAACGGTTGTGCTGTACTCGCTGACGCCGGCATCGCGCTTGCTACTGAAGGATGGTTCTCTCAATACAACGCCATTCCTGCTGATGATCCTGGACCCTGTGGTGACTACACCGTTTCACCTAATGGGGGCCTGGCTGAAAATTAACGGTGGCGACGACCCGGCCGCAACATGCACCCCTTTCGAAATGGAAAATGGTATGCCATTCTGGGAGCTAGGCGCCCGCGAGCCGCGCTTTGGCAACTTATTTAACGAAGCAATGGAAGCCGATTCAAAACTTATTGGCCGCGTCGTTGTCGAGGAATGCGGGGGTGTCTTTGAAGGCTTGAAATCCCTGATGGATGTAGGCGGTGGTAGTGGAACCATGGCGAAAGCAATTGTAAATGCCTTTCCTAATATCAACTGCACAGTGTTCGACCAGCCACATGTGGTTGCCGGCCTGCAGGGTACAACCCACAACCTGGGCTTTATGGGTGGTGATATGTTCGAAGAAATTCCTCCGGCTAACGCCATTCTGCTGAAATGGATTATGCACGATTGGAACGACGCAGAATCTGTTACTATTCTGAAGAAATGTCGTGAGGCGATAAGCCTGTCGAAAAATGAAGGCGGCAATAAAAAGATAATCATTATTGACATCGTAGTCGGGTATGTAGACAACAAGAAAAAGATGATGGATAAGAAAAGCATTGAAACGCAGTTAATGTTTGATATGCTTATGATGAGCATTTTACCGGGAAAAGAACGCAGCAAATCTGAGTGGGAGAAAATCTTCTTTGCAGCAGGTTTCACGCACTACAATATTACCCACACGCTGGGTTTTCGCAGCTTGATTGAAGTCTATCCGTAAttgtagactcggatccaaactcgag |
| MdoOMT1b | gaattcaaaagatctgagtcttgtacaaattataaacccgagctgctggagaataacgtggcctgagcgaggctcatacggaatcggtttactataaccaatagaaggagagtaaaagATGAGCCTGAGCAACGAGGTGGGAGCGACAAGTCACGAGCTCCTGGGCGCGCAAGCGCAACTGTGGAATCATATTTTCCAGTTCATTAACTCTATGTCCTTGAAATGTGCGGTCCAGCTGGGTATAGCGGATGTCATTCACAATCACGGCCAGCCGATTAGTCTGAGTGAGCTGATGGCTGGCCTTAAGGTACATCCGAGTAAAGCTCATTTCGTGAGCCGCCTGATGCGGATTCTGGTCCACAGCAATTTCTTCGCGCAGCATCACCACGTTCACCACGATCGTGCGGATGTGGAAGAAGAAGAAACCGTAGTGCTTTACTCTCTTACGCCGGCGTCCCGTCTGCTGCTGAAAGATGGGAGCCTGAACACGACCCCGTTTCTGCTGATGATTCTGGACCCTGTAGTAACCACCCCGTTCCACCTAATGGGCGCCTGGCTCAAAATCAATGGCGGTGATGATCCTGCGGCAACTTGTACTCCGTTTGAGATGGAGAATGGTATGCCGTTCTGGGAACTTGGTGCAAGAGAGCCTCGCTTCGGCAATCTATTCAATGAAGCAATGGAAGCGGACAGTAAACTGATTGGCCGCGTGGTTGTTGAGGAGTGTGGTGGCGTATTCGAGGGCCTGAAAAGCCTGATGGACGTAGGGGGCGGTTCAGGTACTATGGCCAAAGCGATTGCAAATGCGTTCCCGAACATCAACTGTACCGTCTTCGACCAACCCCACGTCGTGGCAGGATTGCAGGGCACTACTCACAACCTGGGATTCATGGGCGGTGATATGTTTGAAGAGATTCCGCCGGCCAATGCGATTTTACTGAAGTGGATTATGCACGATTGGAACGACGAAGAAAGCGTTACAATTCTCAAGAAATGTCGCGAAGCGATTTCACTGAGCAAGAACGAGGGTGGTAATAAGAAAATCATCATCATAGATATTGTGGTTGGCTACGTAGATAATAAGAAGAAAATGATGGATAAAAAGAGCATCGAAACCCAGCTCATGTTCGATATGTTAATGATGTCTATCCTTCCTGGCAAAGAAAGAAGTAAATCCGAGTGGGAGAAAATTTTCTTTGCCGCCGGTTTCACCCACTACAACATCACCCATACCCTGGGCTTTCGCTCTTTAATTGAAGTGTACCCATGAttgtagactcggatccaaactcgag |
| PhIGS1 | gaattcaaaagatctgagtcttgtagctggagtgtctgtggcaagtccagtgctttgggtctactggtactggaagatcacatgaatagcacataggagattatctttATGACGACAGGCAAAGGCAAAATATTGATATTGGGCGCCACCGGATATCTGGGGAAGTACATGGTGAAAGCTTCTATTTCATTGGGCCATCCGACCTATGCCTACGTGATGCCTTTAAAGAAAAATAGCGATGACAGCAAATTACAGTTGCTGAAAGAGTTTGAAAGCCTGGGTGTTACCATCTTTTACGGCGAACTGTCTGAACACGATAAACTCGTTGCAGTATTCAAGGAAGTGGACATCGTTATTTCTACGCTCGCGGTCCCGCAATACCTTGAACAGCTGAAAGTTATTGAAGCGATTAAAGAAGCCGGAAATATTAAACGTTTCGTTCCGTCGGAATTTGGCAATGAAGTCGATCGTGTCCGCGCGCTTCCGCGCTTCCAGGCCGTATTGGACAACAAGAAAAAGATTCGTCGCGCTACCGAGGCCGCGGGCATTCCGTTTACGTTTGTCAGCGCCAACTCGCTGACAGCCTATTTCGTAGATTACCTGCTGCACCCGCGCCAGAAAAGTGAACAAGTGACTATTTATGGCAGCGGAGATGCGAAAGCGGTTTTAAACTACGAAGAAGACGTTGCCGCGTATACTATTAAAGCCGCAGATGATCCTCGTGCGGCAAATCGCGTACTCATTATTAAACCCCCGAAAAATATTGTGAGCCAGTTGGATCTGGTGAGCAGTTGGGAAAAGACTACAGGTTCCACCCTGAAAATGACGCATATTTCAGAACAAGAAATCATAAAATTGTCGGAGAGCATTAATTTTCCGGAAAATATTCACGCGAGCATACTTCACAATATCTTTATTGCTGGCGCCCAGCTGAGTTTCGAATTGACCCAAGATCATGACCTGGAAGCGTCTGAACTGTATCCAAACTATAATTATACTTCAGTGGATGAATATCTGAAAATTTGCCTTGTGAACCCTCCGAAACCTAAACTGGCCACCTACGCGCAGCCGTCGACATAAttgtagactcggatccaaactcgag |
| CbIGS1 | gaattcaaaagatctgagtcttgtaggccgtagcattataccttgaatagtgtcggagctgcgtttcctcgaaaggtttgaggggagcagacggcgttatttccaaggaggctttgATGGAGAAAATAATTATATACGGGGGTACAGGCTATATTGGTAAATTTATGGTACGCGCGTCGCTGTCATTTTCACATCCGACATTTATATATGCGCGTCCCTTGACGCCTGATAGTACTCCGAGCAGCGTGCAGCTACGTGAAGAATTTCGTTCTATGGGCGTTACCATCATTGAAGGCGAGATGGAAGAACATGAAAAGATGGTTTCAGTGTTACGCCAAGTGGACGTGGTCATATCGGCCCTGTCAGTTCCGATGTATCCGTCGCAGCTGTTAATCATCGATGCCATCAAAGCCGCTGGCAATATTAAGCGCTTTTTACCAAGCGAATTTGGGTCTGAAGAGGATCGCATCAAACCGCTGCCACCATTTGAATCTGTCCTGGAAAAGAAAAGAATTATTCGCCGTGCCATTGAAGCAGCAGAATTACCATACACCTATGTATCGGCCAATTGTTTCGGCGCGTACTTCGTTAACTACCTGTTGCACCCGAGCCCACATCCAAATCGGGATGATGATATTGTTATTTATGGCACTGGCGAAACCAAGTTTGTGCTGAACTATGAGGAGGACATTGCGAAATACACCATTAAGGTGGCCTGCGACCCGAGATGTTGTAACCGTATCGTAATTTACCGCCCGCCGAAGAACATTATTTCGCAGAATGAGCTCATTTCGCTGTGGGAAGCCAAATCAGGCCTGAGCTTTAAGAAAGTCCACATGCCCGATGAGCAGCTCGTCCGTCTGTCTCAGGAGCTCCCCCAACCGCAGAACATTCCGGTGAGCATTCTTCACAGCATCTTTGTAAAGGGCGATCTGATGTCTTACGAAATGCGCAAAGATGATATTGAAGCGTCCAACCTCTACCCGGAATTGGAATTTACCAGCATCGACGGCCTATTGGATCTGTTTATCAGTGGCCGTGCTCCACCGCCTACCCTGGCCGAATTTGAATGAttgtagactcggatccaaactcgag |

**Supplementary Table S5.** Description, catalytic conversion, application, and source of the plasmids used and assembled in this study.

| **Plasmid ID** | **Description** | **Target Conversion** | **Application** | **Reference** |
| --- | --- | --- | --- | --- |
| SBC007589 | pBbAa-1-FjTAL | Tyrosine → Coumaric Acid | Coumaric acid production | [7] |
| SBC010695 | pBbAa-1-SeC3H-PkCOMT-FjTAL | Tyrosine → Ferulic Acid | Ferulic acid production | [8] |
| SBC015863 | pBbAc-1-MsCAD-5-SrCAR-5-BsSfp | Ferulic Acid → Coniferol | Coumarol & Coniferol production | [9] |
|  |  | Coumaric Acidv→ Coumarol |  |  |
| SBC015866 | pBbEc-1-BsSfp-MsCAD-SrCAR | Ferulic Acid → Coniferol | Coumarol & Coniferol production | [9] |
|  |  | Coumaric Acid → Coumarol |  |  |
| SBC015869 | pBbEc-1-MsCAD-1-BsSfp-SrCAR | Ferulic Acid → Coniferol | Coumarol & Coniferol production | [9] |
|  |  | Coumaric Acid → Coumarol |  |  |
| SBC009876 | pBbBk-1-PhCFAT-ObEGS1 | Coniferol → Eugenol | Eugenol & Chavicol production | [7] |
|  |  | Coumarol → Chavicol | Cloning Vector Backbone |  |
| SBC015898 | pBbBk-1-PhCFAT-ObEGS1-EOMT1 | Coniferol → Methyleugenol | Methyleugenol & Methylchavicol production | *This study* |
|  |  | Coumarol → Methylchavicol |  |  |
| SBC015899 | pBbBk-1-PhCFAT-ObEGS1-1-EOMT1 | Coniferol → Methyleugenol | Methyleugenol & Methylchavicol production | *This study* |
|  |  | Coumarol → Methylchavicol |  |  |
| SBC015900 | pBbBk-1-PhCFAT-ObEGS1-IEMT1 | Coniferol → Methyleugenol | Methyleugenol production | *This study* |
| SBC015901 | pBbBk-1-PhCFAT-ObEGS1-1-IEMT1 | Coniferol → Methyleugenol | Methyleugenol production | *This study* |
| SBC015902 | pBbBk-1-PhCFAT-ObEGS1-RcOMT1 | Coniferol → Methyleugenol | Methyleugenol & Methylchavicol production | *This study* |
|  |  | Coumarol → Methylchavicol |  |  |
| SBC015903 | pBbBk-1-PhCFAT-ObEGS1-1-RcOMT1 | Coniferol → Methyleugenol | Methyleugenol & Methylchavicol production | *This study* |
|  |  | Coumarol → Methylchavicol |  |  |
| SBC015904 | pBbBk-1-PhCFAT-ObEGS1-AIMT1 | Coniferol → Methyleugenol | Methyleugenol & Methylchavicol production | *This study* |
|  |  | Coumarol → Methylchavicol |  |  |
| SBC015905 | pBbBk-1-PhCFAT-ObEGS1-1-AIMT1 | Coniferol → Methyleugenol | Methyleugenol & Methylchavicol production | *This study* |
|  |  | Coumarol → Methylchavicol |  |  |
| SBC015906 | pBbBk-1-PhCFAT-ObEGS1-MdoOMT1aΔ | Coniferol → Methyleugenol | Methyleugenol & Methylchavicol production | *This study* |
|  |  | Coumarol → Methylchavicol |  |  |
| SBC015907 | pBbBk-1-PhCFAT-ObEGS1-1-MdoOMT1aΔ | Coniferol → Methyleugenol | Methyleugenol & Methylchavicol production | *This study* |
|  |  | Coumarol → Methylchavicol |  |  |
| SBC015908 | pBbBk-1-PhCFAT-ObEGS1-MdoOMT1b | Coniferol → Methyleugenol | Methyleugenol & Methylchavicol production | *This study* |
|  |  | Coumarol → Methylchavicol |  |  |
| SBC015909 | pBbBk-1-PhCFAT-ObEGS1-1-MdoOMT1b | Coniferol → Methyleugenol | Methyleugenol & Methylchavicol production | *This study* |
|  |  | Coumarol → Methylchavicol |  |  |
| SBC015910 | pBbBk-1-PhCFAT-PhIGS1 | Coniferol → Isoeugenol | Isoeugenol production | *This study* |
| SBC015911 | pBbBk-1-PhCFAT-CbIGS1 | Coniferol → Isoeugenol | Isoeugenol production | *This study* |

**Supplementary Table S6.** List of chemical standards and substrates used in this study.

| **Compound** | **CAS number** | **Molecular weight**  **(g mol^-1^)** | **Supplier** | **Supplier code** | **Application** | **Quantification method** |
| --- | --- | --- | --- | --- | --- | --- |
| _L_-Tyrosine | 60-18-4 | 181.19 | Acros Organics | 140641000 | Analytical standard & enzyme substrate | LC-QQQ |
| *Trans*-Ferulic acid | 537-98-4 | 194.18 | Sigma Aldrich | 102162380 | Analytical standard & enzyme substrate | UPLC-DAD |
| *p*-Coumaric acid | 501-98-4 | 164.16 | Sigma Aldrich | 102172008 | Analytical standard & enzyme substrate | UPLC-DAD |
| _L-_Methionine | 63-68-3 | 149.21 | Sigma Aldrich | 1002056646 | Substrate (methyl group donor) | N/A |
| IPTG (dioxane free) | 367-93-1 | 238.3 | Formedium | IPTG025 | Substrate (promoter inducer) | N/A |
| *Sec*-butylbenzene | 135-98-8 | 134.22 | Sigma Aldrich | 102312489 | Internal Standard | GCMS |
| Eugenol | 97-53-0 | 164.2 | Acros Organics | 119110050 | Analytical standard | GCMS |
| Isoeugenol | 97-54-1 | 164.2 | Acros Organics | 122570050 | Analytical standard | GCMS |
| 3-(4-Hydroxyphenyl)-1-propene (Chavicol) | 501-92-8 | 134.18 | Fluorochem | 398756 | Analytical standard | GCMS |
| 4-Allyl-1,2-dimethoxybenzene (Methyleugenol) | 93-15-2 | 178.23 | Sigma Aldrich | 1003109143 | Analytical standard | GCMS |
| 4-Allylanisole (Methylchavicol) | 140-67-0 | 148.2 | Acros Organics | 102880050 | Analytical standard | GCMS |

#
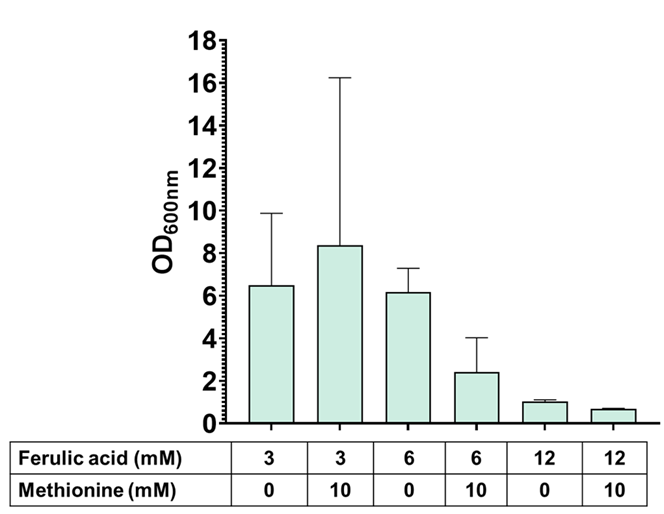
Supplementary Figures

**Supplementary Figure S1.** OD_600nm_ readings of strain SKF100_001080 after 24 hours growth in conditions of increasing concentrations of ferulic acid (mM) and methionine (mM). All strains were grown in the presence of IPTG, and measurements were taken 24 hours after induction and addition of the appropriate substrates. Error bars are representative of standard deviations of biological triplicates.

**
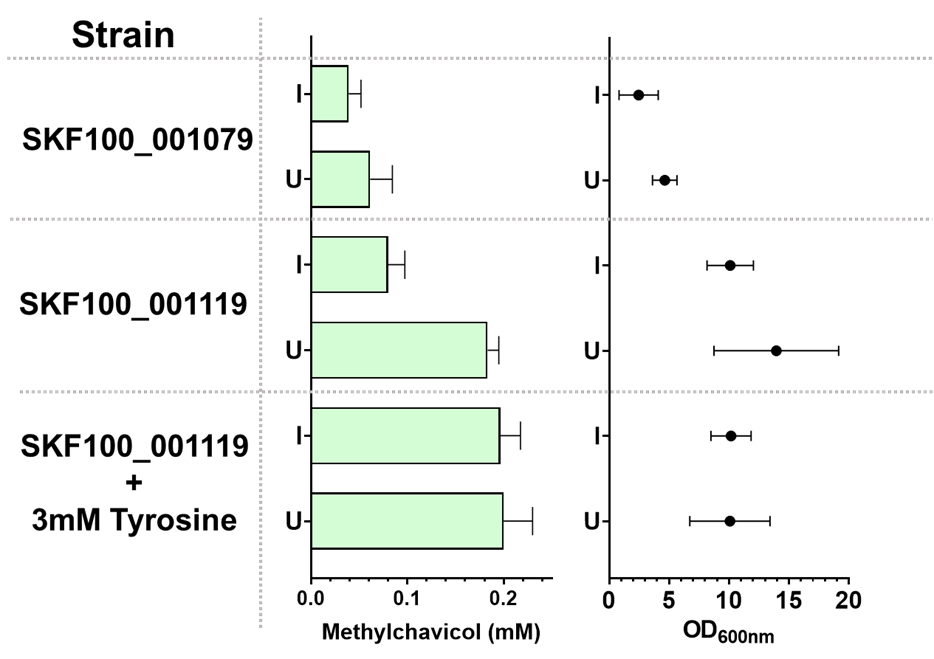
**

**Supplementary Figure S2.** Methylchavicol titres and corresponding OD_600nm_ readings of strains SKF100_001079 and SKF100_001119 after 24-hour production assay. All strains were grown in the absence (U) or presence (I) of IPTG, and measurements were taken 24 hours after induction and addition of the appropriate substrates. Error bars are representative of standard deviations of biological triplicates.


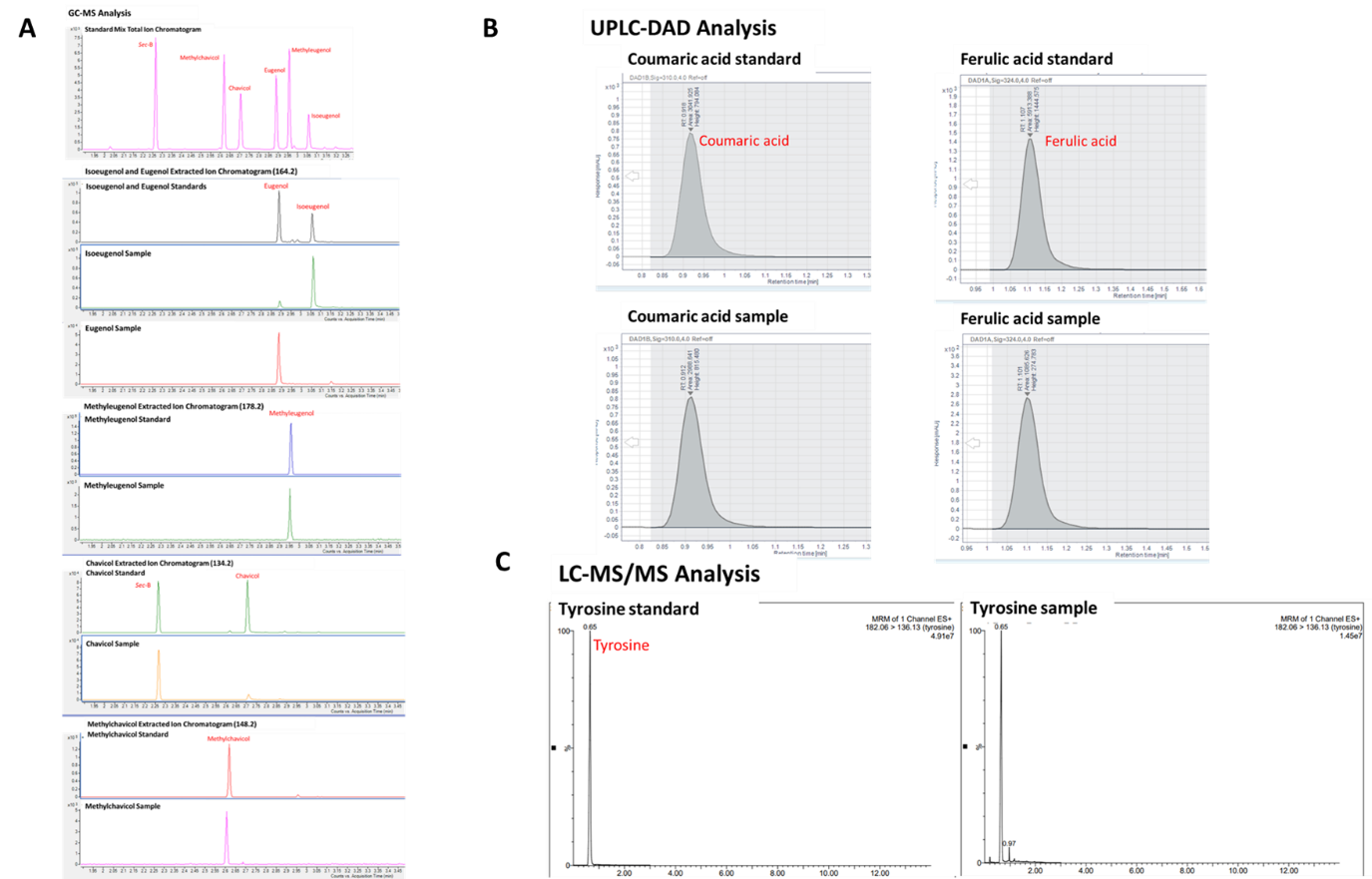


**Supplementary Figure S3.** Chromatographs of the target compounds and their respective standards. **A.** Chromatographs of GC-MS target compounds: isoeugenol, eugenol, methyleugenol, chavicol and methylchavicol. **B.** Chromatographs of UPLC-DAD targets: coumaric acid and ferulic acid. **C.** Chromatographs of LC-MS/MS target: tyrosine.

# References

1. Gang, D.R., et al., *Characterization of phenylpropene O-methyltransferases from sweet basil: facile change of substrate specificity and convergent evolution within a plant O-methyltransferase family.* Plant Cell, 2002. **14**(2): p. 505-519, DOI: 10.1105/tpc.010327

2. Wang, J. and E. Pichersky, *Characterization of S-Adenosyl-l-methionine:(Iso) eugenol O-methyltransferase involved in floral scent production in Clarkia breweri.* Arch. Biochem. Biophys., 1998. **349**(1): p. 153-160, DOI: 10.1006/abbi.1997.0452.

3. Wu, S., et al., *Two O-methyltransferases isolated from flower petals of Rosa chinensis var. spontanea involved in scent biosynthesis.* J. Biosci. Bioeng., 2003. **96**(2): p. 119-128, DOI: 10.1016/S1389-1723(03)90113-7

4. Koeduka, T., et al., *Biosynthesis of t-anethole in anise: characterization of t-anol/isoeugenol synthase and an O-methyltransferase specific for a C7-C8 propenyl side chain.* Plant Physiol., 2009. **149**(1): p. 384-94, DOI: 10.1104/pp.108.128066

5. Yauk, Y.K., et al., *The O-methyltransferase gene MdoOMT1 is required for biosynthesis of methylated phenylpropenes in ripe apple fruit.* Plant J., 2015. **82**(6): p. 937-950, DOI: 10.1111/tpj.12861

6. Koeduka, T., et al., *The multiple phenylpropene synthases in both Clarkia breweri and Petunia hybrida represent two distinct protein lineages.* Plant J., 2008. **54**(3): p. 362-374, DOI: 10.1111/j.1365-313X.2008.03412.x

7. Robinson, C.J., et al., *Rapid prototyping of microbial production strains for the biomanufacture of potential materials monomers.* Metab. Eng., 2020. **60**: p. 168-182, DOI: 10.1016/j.ymben.2020.04.008

8. Dunstan, M.S., et al., *Engineering Escherichia coli towards de novo production of gatekeeper (2S)-flavanones: naringenin, pinocembrin, eriodictyol and homoeriodictyol.* Synth. Biol. (Oxford, U. K.), 2020. **5**(1), DOI: 10.1093/synbio/ysaa012

9. Hanko, E.K.R., et al., *Carboxylic acid reductase-dependent biosynthesis of eugenol and related allylphenols.* PREPRINT (Version 1) available at Research Square, 2023, DOI: 10.21203/rs.3.rs-3309580/v1
